# FePc and FePcF_16_ on Rutile TiO_2_(110) and (100): Influence of the Substrate Preparation on the Interaction Strength

**DOI:** 10.3390/molecules24244579

**Published:** 2019-12-13

**Authors:** Reimer Karstens, Mathias Glaser, Axel Belser, David Balle, Małgorzata Polek, Ruslan Ovsyannikov, Erika Giangrisostomi, Thomas Chassé, Heiko Peisert

**Affiliations:** 1 Institute of Physical and Theoretical Chemistry, University of Tübingen, Auf der Morgenstelle 18, 72076 Tübingen, Germany; Nikolaus-Reimer.karstens@uni-tuebingen.de (R.K.); mathias_glaser@gmx.de (M.G.); axel.belser@uni-tuebingen.de (A.B.); david.balle@uni-tuebingen.de (D.B.); malgorzata.polek@uni-tuebingen.de (M.P.); Thomas.Chasse@uni-tuebingen.de (T.C.); 2 Institute for Methods and Instrumentation in Synchrotron Radiation Research, Helmholtz-Zentrum Berlin für Materialien und Energie GmbH, Albert-Einstein-Straße 15, 12489 Berlin, Germany; ovsyannikov@helmholtz-berlin.de (R.O.); erika.giangrisostomi@helmholtz-berlin.de (E.G.); 3 Center for Light-Matter Interaction, Sensors & Analytics (LISA+) at the University of Tübingen, Auf der Morgenstelle 18, 72076 Tübingen, Germany

**Keywords:** rutile, iron phthalocyanine, interfaces, photoemission, X-ray absorption

## Abstract

Interface properties of iron phthalocyanine (FePc) and perfluorinated iron phthalocyanine (FePcF_16_) on rutile TiO_2_(100) and TiO_2_(110) surfaces were studied using X-ray photoemission spectroscopy (XPS), X-ray absorption spectroscopy (XAS), and low-energy electron diffraction (LEED). It is demonstrated that the interaction strength at the interfaces is considerably affected by the detailed preparation procedure. Weak interactions were observed for all studied interfaces between FePc or FePcF_16_ and rutile, as long as the substrate was exposed to oxygen during the annealing steps of the preparation procedure. The absence of oxygen in the last annealing step only had almost no influence on interface properties. In contrast, repeated substrate cleaning cycles performed in the absence of oxygen resulted in a more reactive, defect-rich substrate surface. On such reactive surfaces, stronger interactions were observed, including the cleavage of some C–F bonds of FePcF_16_.

## 1. Introduction

The surface science of titanium dioxide is widely investigated, driven by a broad variety of applications found in many fields [1]. As an example, TiO_2_ in different forms is included in photovoltaics, well-established devices are TiO_2_-based dye sensitized solar cells (DSSC) [2,3,4]. Good efficiencies of DSSCs have been obtained by the use of transition metal phthalocyanines (TMPcs), including iron phthalocyanine (FePc) as the light harvester [5,6]. Although numerous applications are based on titanium dioxide nanomaterials [7,8,9], well defined single crystalline rutile or anatase TiO_2_ surfaces can serve as a model system.

Nevertheless, investigations of interface properties between TMPcs and TiO_2_ are comparably rare and different results about the interaction strength are reported. As an example, the deposition of CoPc on rutile TiO_2_(110) at room temperature results in mobile molecules, only weakly interacting with the substrate (physisorption) [10]. In contrast, a rather strong interaction for FePc and CuPcF_16_ was observed on the same substrate surface [11,12], which, in the case of FePc, may even include a chemical reaction of the phthalocyanine macrocycle [11]. For applications, the aim is not necessarily to suppress interactions at interfaces, but advantageous properties may arise from a strong electronic coupling: The interaction between CuPcF_16_ and TiO_2_(110) enables an ultrafast charge transfer from CuPcF_16_ molecules to the TiO_2_ substrate on the time scale of 10 fs [12].

**Figure 1:**
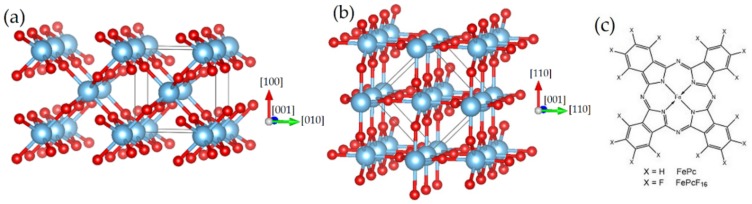
Bulk-terminated crystal structure of rutile TiO_2_ (**a**) (100) and (**b**) (110) surface. (**c**) Chemical structure of studied iron phthalocyanines.

Due to the complex surface chemistry of TiO_2_ surfaces [1], it might be expected that the detailed preparation conditions significantly affect the interaction strength between molecules and the substrate. Our studies aim to a more systematic understanding of the interface properties of TMPc/rutile interfaces. Generally, different interaction channels are observed for TMPcs on different substrates, involving both the central metal atom and the macrocycle [13]. Here, we focus on interface properties between iron phthalocyanine (FePc) and perfluorinated iron phthalocyanine (FePcF_16_) on rutile TiO_2_(100) and compare selected results to rutile TiO_2_(110). The interfaces were studied using X-ray photoemission spectroscopy (XPS), X-ray absorption spectroscopy (XAS), and low-energy electron diffraction (LEED). It was reported that the rutile TiO_2_(100) surface is more reactive compared to rutile TiO_2_(110), since, for example, water dissociation is observed on rutile TiO_2_(100) surfaces, whereas most studies agree that on rutile TiO_2_(110) surfaces, such a chemical reaction can occur only at oxygen vacancies [14,15,16]. The fluorination of FePc results in a higher ionization potential, which may increase the ability for an electron transfer to the molecule at the interface, similar to CoPc/CoPcF_16_ [17]. The crystal structures of the studied rutile surfaces are shown in Figure 1, together with the chemical structure of the molecules.

## 2. Results and Discussion

### 2.1. Characterization of the Substrate Surfaces

The detailed atomic composition and arrangement of the substrate surface may affect the reactivity significantly, and thus, differences between rutile TiO_2_(100) and rutile TiO_2_(110) might be expected. On the other hand, the crystal preparation may influence the number of defects, and thus the reactivity of the substrate surface. Thus, the surface structure has to be accounted for, including surface roughness and reconstructions. Further, distinguishing between the number of defects present on the surface and in the bulk of the crystal is important.

After checking the cleanliness of the rutile crystal following the sputtering/annealing cycles, we quantified the number of total defects via Ti 2p photoemission spectra. The defect chemistry of TiO_2_ is very complex, among other induced defects include oxygen vacancies and (metal) ions in the interstitial sites [18]. Reduced titanium ion species, such as Ti^3+^, are visible in Ti 2p photoemission spectra as an additional peak or shoulder at the low binding energy side.

**Figure 2:**
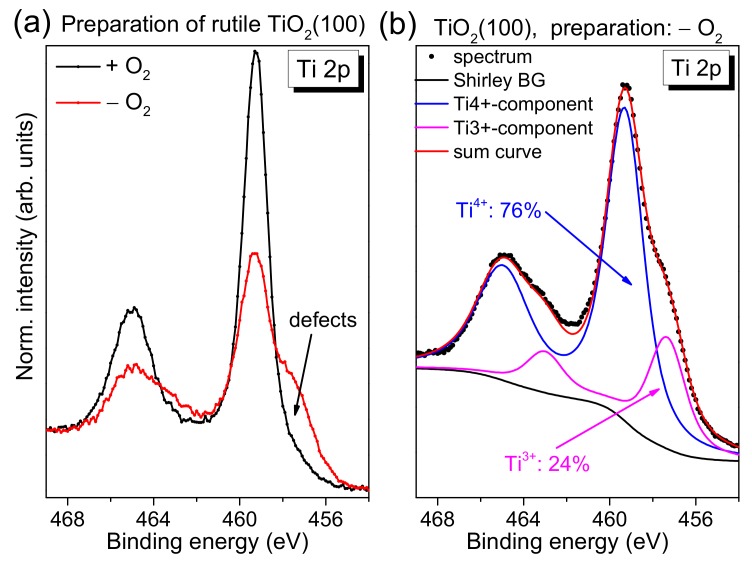
Typical Ti 2p spectra (hν = 1486.6 eV) of differently prepared rutile crystals (in the presence and absence of oxygen during the last annealing step). The low binding energy component can be essentially assigned to Ti^3+^. (**a**) Effect of the different preparation on the Ti^3+^ intensity. (**b**) Exemplary peak fits are shown for rutile TiO_2_(100).

In Figure 2a, we compare typical Ti 2p spectra of rutile TiO_2_(100) for two different preparation methods. Conventional Al Kα radiation (hν = 1486.6 eV) was used for excitation. The preparation was performed in the presence and absence of an oxygen partial pressure during the last annealing step, denoted “+O_2_” and “−O_2_”, respectively. Clearly visible, the shoulder at the low binding energy side is significantly increased for the preparation in UHV, indicating a higher number of defects. Generally, due to a preferred removal of oxygen during the sputtering process, defects are created. Diffusion of oxygen from the bulk to the surface of the substrate during the annealing steps can—to some extent—compensate surface defects, while leaving defects in the bulk. As a consequence, non-stoichiometric surfaces are obtained (TiO_x_, x < 2). The presence of such point defects can be formally assigned to the presence of a Ti^3+^ oxidation state in the Ti 2p photoemission spectrum [1]. The nature of such defect states is explained by the removal of negative oxygen ions from the lattice as neutral oxygen atoms, leaving electrons in the conduction band [1]. Apparently, the annealing in oxygen partial pressure during all preparation steps supports the filling of oxygen vacancies by adsorption and diffusion, enabling the preparation of an almost defect-free substrate (cf. Figure 2a, black curve). We note that the longer the substrate is exposed to sputtering/annealing cycles, the more defects are created in the bulk of the material, which cannot be removed completely by exposure to an oxygen partial pressure during the annealing steps due to the limited oxygen diffusion to the surface. In this case, also the exposure to oxygen during the last annealing step is not sufficient to remove all defects. This results in a remaining intensity in Ti 2p spectra at the position of defects, although the quality of the substrate surface is almost independent of the previous treatment of the crystals, as indicated by complementary methods (see below).

In order to estimate the number of total defects, peak fits to the Ti 2p spectra were performed as exemplarily, shown for defect-rich rutile TiO_2_(100) in Figure 2b. The spectrum can be essentially described by two doublets. The main Ti 2p_3/2_ component is found at 459.4 ± 0.1 eV and can be ascribed to Ti^4+^ species, in good agreement with the literature [1,19,20]. The second doublet at about 1.9 ± 0.1 eV lower binding energy arises essentially from a Ti^3+^ species [1,19,20,21,22]. Contributions from Ti^2+^, expected at 3.0–3.4 eV lower binding energy with respect to the main line [19,21,22], are generally weak and were neglected in the description of the Ti 2p spectra (except for defect-rich, hexagonal rutile TiO_2_(100), discussed in Section 2.5). Detailed fitting parameters can be found as a Supporting Information (Table S1). As clearly visible from Figure 2b, the absence of O_2_ during the last annealing step can result in a high number of defects, visible as a high Ti^3+^ intensity (24% in Figure 2). Such defects may act as reactive sites on the crystal surface. However, for the reactivity of the substrate surface, it is essential whether the defects are preferably located in the bulk of the material or at the surface. Energy-dependent photoemission studies (i.e., changing the surface sensitivity) for substrates with a higher Ti^3+^ intensity in Ti 2p spectra indicate that the majority of the defects is found in the bulk of the rutile substrate (Figure S1 and Tables S2 and S3).

Additionally, LEED was performed routinely to characterize the substrate surfaces, giving further hints for the presence of defects in the topmost layer(s); examples are shown in Figure S2. The rutile TiO_2_(100) and TiO_2_(110) surfaces prepared under oxygen show typical 1 × 1 LEED patterns. Samples prepared without oxygen in the last annealing step exhibit a brighter background and additional streaks, indicating the presence of smaller well-ordered domains at the surface and/or the presence of additional ordered structures on the substrate surface.

**Figure 3:**
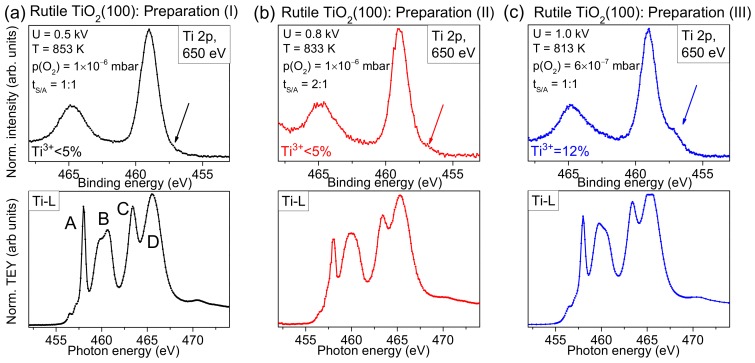
Different preparation of rutile TiO_2_(100): Ti 2p spectra (top panels) and Ti-L edges (bottom panels). The lowest sputter voltage U and the annealing temperature of 853 K results in an almost defect free rutile TiO_2_(100) in presence of O_2_ (partial pressure p(O_2_) = 1 × 10^–6^ mbar) during annealing. For (**a**,**c**), the same sputtering to annealing time ratio t_S/A_ was applied (30 min each). In contrast, for (**b**) a sputtering time of 40 min and an annealing time of 20 min was chosen.

Complementary, a detailed analysis of Ti-L edge XAS spectra is useful for the characterization of the substrate surface. The method allows, in particular, the determination of polymorphs and the degree of order of the investigated substrates within the information depth of about 10 nm [23,24]. In Figure 3, we compare Ti-L edge XAS spectra with surface sensitive Ti 2p XPS spectra (hυ = 650 eV) for three different preparations, marked with roman numerals (I), (II), and (III). The parameters for preparation (I) were used for experiments described in Section 2.2, Section 2.3 and Section 2.4. For preparations (II) and (III), the sputtering voltage was successively increased and the annealing temperature decreased. Thus, a larger structural damage by ion bombardment and a lower ability to recover the crystal structure due to the lower oxygen partial pressure, and a reduced thermal diffusion of oxygen ions in the crystal might be expected. Detailed preparation parameters are given in Figure 3.

The XPS spectrum in Figure 3a exhibits only weak intensity in the energy region of Ti^3+^ (see arrow), and the corresponding XAS spectrum reproduces well the expected features of a rutile single crystal: Features A–D are clearly resolved. The nature of transitions and the shape of different Ti-L XAS spectra is described in detail in [23,24]. Although for preparation (II) only very weak intensity in the Ti^3+^ region of the XPS spectrum is detected in Figure 3b, the Ti-L edge XAS spectrum seems to be more sensitive and indicates imperfections in the crystal: The relative intensity of feature A is decreased and the fine structure of feature B is lost. In addition, all features are broadened. Finally, for preparation (III), prepared using the highest sputter voltage and lowest annealing temperature, a noticeable intensity in the energy range of Ti^3+^ is already visible in the surface sensitive Ti2p spectrum (see arrow in Figure 3c). This indicates that the preparation conditions are not sufficient to saturate the defects at the surface, although the shape of some XAS features appear more similar to preparation (I) (Figure 3a) than to preparation (II) (Figure 3b). Thus, the examples in Figure 3 indicate that minor changes of preparation conditions may result in very different surface structures, which might explain different interface properties between organic molecules and rutile reported in the literature (see below).

### 2.2. FePc on Rutile TiO_2_(100)

**Figure 4:**
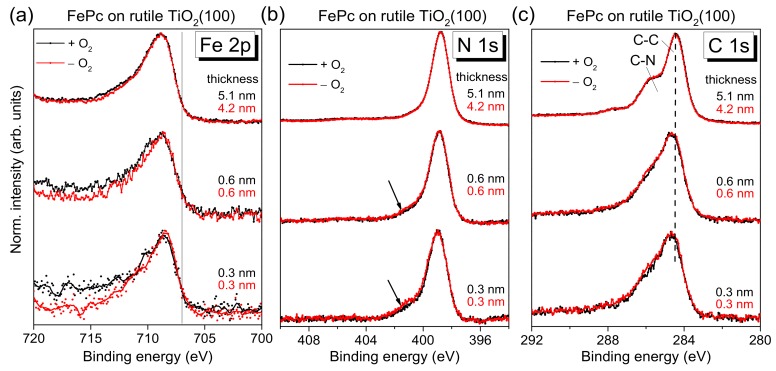
Thickness-dependent core-level spectra of FePc on rutile TiO_2_(100) (hυ = 1486.6 eV): Comparison of different substrate preparations. (**a**) Fe 2p, (**b**) N 1s, (**c**) C 1s. The exposure to oxygen in the last annealing step does not significantly affect the spectral shape. At low coverages, additional intensity is visible in N 1s spectra (arrows) and Fe 2p spectra (black line). C 1s spectra are distinctly broadened compared to the bulk-like FePc films of 4–5 nm.

In the following, we discuss how the different preparations of substrate surfaces affect the interaction with organic molecules at the interface. First, we discuss FePc on rutile TiO_2_(100). The corresponding XPS core-level spectra of the organic molecule are shown in Figure 4 as a function of the film thickness. No significant change of both the peak position and the peak shape of substrate-related core-level peaks (O 1s and Ti 2p, not shown) was visible upon FePc deposition. Black and red curves in Figure 4 correspond to FePc deposited on rutile TiO_2_(100) prepared in the presence and absence of O_2_ partial pressure during the last annealing step. The shapes of the spectra for the thickest films are typical for (iron) phthalocyanines and in excellent agreement to the literature [25,26,27,28,29,30]. The Fe 2p spectra show an intensity maximum at a binding energy (BE) of 708.8 eV and exhibit the typical multiplet structure for Fe in FePc (e.g., [25,26,27]). The detailed shape of N 1s and C 1s spectra is described below more in detail based on the respective peak fits. Most important, the black and red curves in Figure 4 are very similar for all core levels, implying that the presence of oxygen during the last annealing step does not affect distinctly the number of reactive defects at the substrate surface. In other words, the absence of oxygen in the last annealing step and the possible formation of surface defects can be obviously compensated, to a large extent, by diffusion of oxygen from deeper layers of the substrate.

On the other hand, the core-level spectra of Figure 4 exhibit some thickness-dependent changes, which we discuss in turn below. First, the Fe 2p spectra appear slightly broadened for low coverages, but the complex multiplet structure complicates a more detailed analysis by a peak fitting routine. Therefore, we discuss the thickness dependence of the Fe 2p spectra in Figure 4a later, together with the corresponding XAS data.

**Figure 5:**
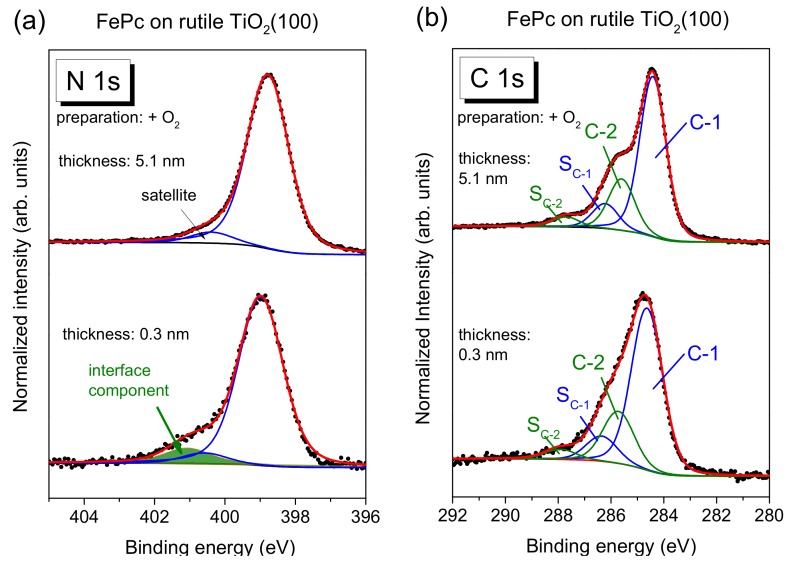
Exemplary peak fits of N 1s (**a**) and C 1s (**b**) core-level spectra of FePc on rutile TiO_2_(100) (hυ = 1486.6 eV). Whereas for low coverages, an interface component has to be introduced for N 1s, a broadening is sufficient for C 1s.

The N 1s core-level spectra for low coverages in Figure 4b show clearly additional intensity at higher binding energies (see arrows) compared to the bulk-like references (the 4–5 nm thick films). In order to quantify this additional intensity, we performed peak fits to the data, shown in Figure 5a, exemplary for the rutile preparation in presence of oxygen during the last annealing step.

The main line of the N 1s spectrum contains contributions from two types of chemically inequivalent nitrogen atoms in the FePc molecule (cf. Figure 1c), which cannot be resolved experimentally due to their small separation of about 0.3 eV [26]. The main line is accompanied by weaker shake-up satellite features, the most intense satellite appears at about 1.7 eV higher binding energy (e.g., [26,28]). For the bulk-like, 5.1 nm thick FePc film on rutile TiO_2_(100) prepared in the presence of oxygen during the last annealing step, we find the peak maximum of the main component at 398.8 eV and the satellite at BE = 400.4 eV. At the interface, the N 1s spectrum is shifted slightly by 0.2 eV towards higher binding energies, and the additional intensity can be described by a single peak at 2.05 eV higher binding energy with respect to the main peak. The relative intensity of the interface component is about 7%. In this fitting model, a change of the satellite intensity at the interface is neglected; a distinct suppression is, in particular, observed for organic molecules on metal surfaces [11,31,32,33]. This low relative intensity of the interface component may indicate that only few molecules at the interface become affected by an interaction involving the nitrogen atoms (e.g., molecules adsorbed at defect sites), and/or that a local interaction occurs involving particular nitrogen atoms of FePc.

The C 1s spectra of phthalocyanines can be described by components attributed to the aromatic carbon of the benzene rings (C-1), pyrrole carbon linked to nitrogen (C-2) and corresponding satellites (S_C-1_ and S_C-2_) [29,30]. For the FePc film of 5.1 nm in Figure 5b, we obtained binding energies of 284.4 and 285.6 eV for C-1 and C-2, respectively. Detailed fitting parameters can be found in the Supporting Material (Table S4). For the low coverage, the main peak is shifted to 0.2 eV higher binding energy, similar to N 1s. Many reasons might cause such small, rigid shifts of all core levels, among other band bending like effects due to induced density of interface states or a different screening of the photohole in the films of different thickness [34,35,36]. Most important, for the description of the C 1s spectra, no additional component is needed at the interface. The spectrum can be described by a Gaussian broadening of all components (from 1.02 to 1.26 eV) and a decrease of the C-1 to C-2 distance from 1.2 to 1.1 eV. The distinct broadening of all spectra at lower film thickness may indicate different environments of the FePc molecules, such as different adsorption sites, whereas the decrease of the C-1 to C-2 distance might be caused by site-dependent screening [37] or a different charge distribution within the FePc molecule. The detailed peak fit parameters are in the Supplementary Materials (Table S4). In addition, we show as Supplementary Materials peak fits for the rutile preparation in the absence of oxygen during the last annealing step (Figure S3 and Table S5), indicating a similar behavior of FePc on both surfaces.

We note that stronger interactions between FePc and TiO_2_ were reported in the literature [11]. For a differently prepared FePc/rutile TiO_2_(110) interface, the description of the 1–2 monolayer C 1s spectrum was only possible if separate contributions from the first and second layer were considered [11]. In addition, a very strong interface peak at 400.4 eV was observed in the N 1s spectra, which even dominates the N 1s spectrum of the (sub-)monolayer [11]. The shift of the C 1s spectrum of the first layer to 1.2 eV higher binding energy was interpreted as oxidation of the FePc at the interface as a consequence of a surface bond [11]. Since such additional intensity at higher binding energy is not observed in the C 1s spectra of Figure 4 and Figure 5, the nature of the interaction of FePc on the studied rutile TiO_2_(100) interface seems to be different in our case. The question arises if the surface orientation and/or the substrate preparation may be responsible for such differences—this is discussed in Section 2.3 and Section 2.4.

**Figure 6:**
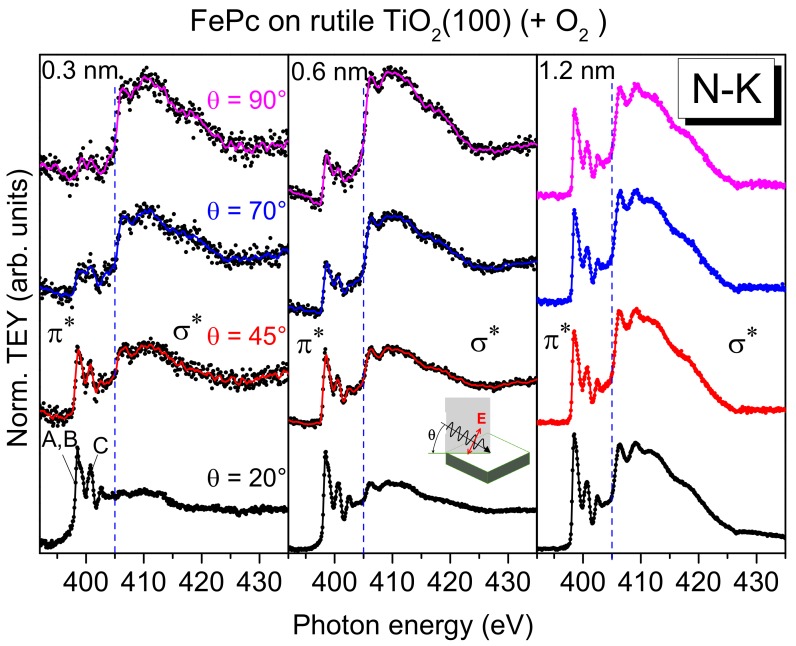
N-K edge X-ray absorption spectra of FePc on rutile TiO_2_(100) prepared in the presence of oxygen during the last annealing step. With increasing film thickness, a weaker anisotropy is observed, indicating a change of the molecular orientation. The smaller relative intensity of the π* transition A for the coverage of 0.3 nm points to a different (unoccupied) electronic structure at the interface. The measurement geometry is shown as an inset (middle panel).

First, we look at the electronic structure of the nitrogen atoms in FePc at the interface to rutile TiO_2_(100) in more detail. Additional information about the involvement of particular atoms in an interaction at interfaces can be gained from XAS. Since XAS provides information about both the (unoccupied) electronic structure and the molecular orientation, we discuss first effects of the molecular orientation on the spectral shape. Polarization dependent N-K edge XAS spectra of FePc films of different thickness on rutile TiO_2_(100) are shown in Figure 6.

The excitation probability for a transition from the N 1s (or C 1s) orbital into either a π^*^-orbital or a σ^*^-orbital is angular-dependent for planar π-conjugated molecules, since the transition dipole moment for excitations into π^*^-orbitals is oriented perpendicular to the molecular plane, while those for σ^*^-orbitals are in the molecular plane (parallel to chemical bonds). Thus, for flat lying (face-on), planar molecules, the strongest excitations into π^*^-orbitals are expected at grazing incidence of the p-polarized synchrotron light (i.e., for the electric field vector of the synchrotron light parallel to the transition dipole moment). In order to gain information on the molecular orientation, the sample was measured at several different incidence angles of the p-polarized light with respect to the substrate surface. The angle was varied between 20° (grazing incidence) and 90° (normal incidence).

At low coverages (0.3 and 0.6 nm), the spectra at normal emission in Figure 6 are dominated by excitations into σ^*^-orbitals (photon energies >405 eV), whereas at grazing incidence, the strongest transitions are observed into π^*^-orbitals (photon energies <405 eV). This indicates a preferred flat lying orientation of the FePc molecules, enabling a maximal interaction of the π-conjugated system with the substrate surface. Such growth modes were observed for low coverages in the monolayer range, if the molecule–substrate interaction is stronger than the molecule–molecule interaction [38]. In contrast, the N-K edge spectra of the 1.2 nm thick FePc film are almost independent of the angle of incidence of the synchrotron light: Both excitations into σ^*^- and π^*^-orbitals are visible. This indicates a change of the molecular orientation with increasing thickness. We note, that N-K XAS spectra of thicker FePc films on rutile TiO_2_(110) show even an anisotropy, where spectra at grazing incidence are dominated by σ^*^-excitations, while the spectra at normal incidence are dominated by π^*^-excitations, pointing to a change of the molecular orientation from preferred flat lying to preferred standing (Figure S4). Such a change of the molecular orientation as a consequence of the interplay between molecule–substrate and molecule–molecule interactions was observed for related phthalocyanines on substrates where atomically flat terraces are small or on oxidic substrates (with supposed smaller surface energies), including TiO_2_ [38,39].

**Figure 7:**
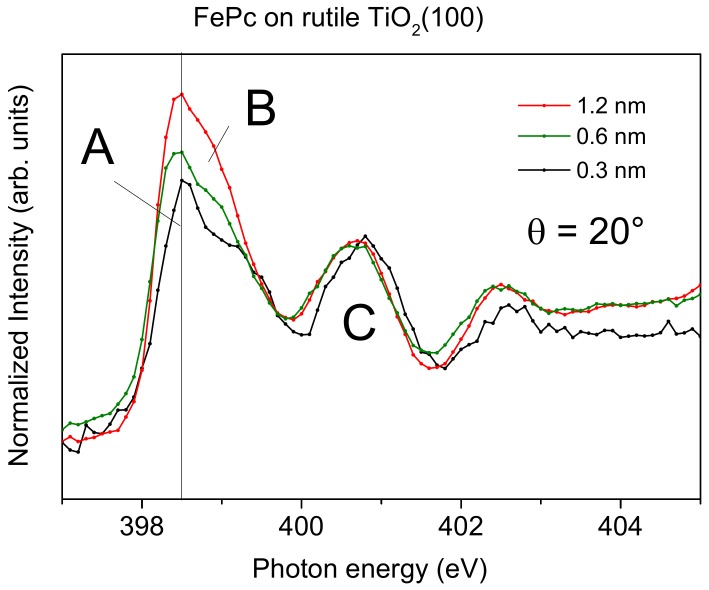
N-K edge X-ray absorption spectra of FePc on rutile TiO_2_(100). Zoom into the region of transitions into the LUMO (A, B, C) as a function of thickness at grazing incidence (θ = 20°). Spectra are normalized to the same intensity of feature C.

However, not only the (relative) intensity of transitions into π^*^- and σ^*^-orbitals change in the N-K XAS spectra of Figure 6. The N-K XAS spectra for the bulk-like 1.2 nm thick FePc film exhibit the typical shape observed for many phthalocyanines. The most intense π^*^ resonances at the lowest photon energies can be assigned to transitions from N 1s to LUMO e_g_ orbitals [40] (denoted A, B in Figure 6). The hybridization with the central metal, most evident for transition metals with not fully occupied lower d-levels (e_g_), causes a distinct splitting into two features A and B (cf. Figure 7), involving excitations from the two inequivalent nitrogen atoms [13,40,41]. Also, the feature C at higher photon energies in Figure 6 can be essentially assigned to transitions into π^*^ states [40]. Since all features, A, B, and C in Figure 6, are related to transitions into the LUMO orbital, only minor changes of the relative intensities are expected as a function of the angle of the incoming synchrotron light and/or the film thickness. However, as clearly visible in Figure 6, the relative intensity of feature A appears to be decreased for low coverages compared to the 1.2 nm thick film. This becomes more evident in Figure 7, where we zoom into the region of LUMO transitions (A, B, C) for films of different thicknesses measured at grazing incidence (with strongest π^*^ resonances for flat lying molecules). Normalizing the data to the same intensity of feature C, one can clearly see the decreased relative intensity of A and B with respect to C for decreasing coverages.

**Figure 8:**
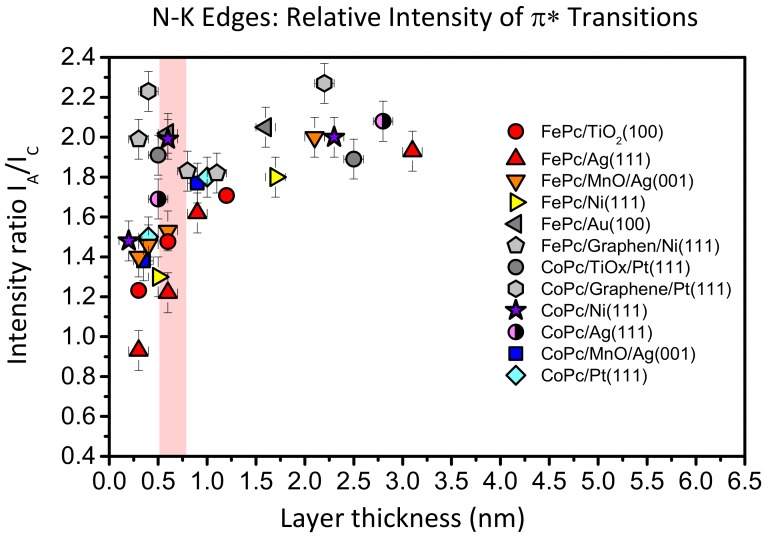
Comparison of relative intensities of transitions into π^*^-orbitals in N-K edge X-ray absorption spectra of FePc and CoPc and on several substrate surfaces as a function of the film thickness. Data from [13,25,27,42].

Such a behavior is not unique for FePc on TiO_2_(100); it was also observed for CuPcF_16_ on TiO_2_ and ascribed to a deformation of the molecules at the interface [12]. In Figure 8, we compare the intensity ratio between features A and C of the N-K absorption spectra of FePc and CoPc on several substrates as a function of the film thickness. The data were taken from [13,25,27,42,43]. Like in Figure 7, we analyzed spectra recorded at grazing incidence, since for most systems at this geometry, the N 1s–π^*^ transitions are strongest due to the preferred flat lying molecular orientation on the substrate surfaces. For bulk-like, more than 1.5 nm thick films, the intensity ratio A:C (peak height) is close to 2:1 and the general shape of the spectra is always similar [13,26,41]. We note that the absolute values of the A:C ratio in the bulk is not necessarily exactly the same for different metal phthalocyanines and different orientations, e.g., due to a different A–B splitting as a result of the hybridization with orbitals from the central metal atom [13] or the presence of a weak, in-plane polarized transitions in the same energy range as the π^*^ resonances [44,45]. However, for many systems shown in Figure 8, the intensity ratio A:C in N-K XAS spectra decreases with decreasing film thickness. The strongest variation is visible for FePc/Ag (111) (red triangles), an interface which exhibits very strong interactions, including a hybridization of Fe and substrate-derived states at the interface [25]. The data for FePc on TiO_2_(100) also follow this trend (red circles in Figure 8): The A:C intensity ratio for the lowest coverage (0.3 nm) is as low as 1.2:1. On the other hand, there are interfaces where the A:C intensity ratio is almost non-dependent on the film thickness (grey data points in Figure 8), pointing to relatively inert interfaces.

Thus, a decreased relative intensity of the feature A seems to be an indication for an enhanced interaction at the interface. For such interfaces discussed in Figure 8, a (partial) charge transfer to the molecule was reported. Therefore, it seems likely that the decrease of the relative intensity of A can be explained by a partial filling of the related orbitals, which reduces the possibility of excitations into these orbitals. We note that such a behavior was also observed for potassium intercalated FePc, where an electron transfer from K to FePc is evident [41]. However, such charge transfer processes are not necessarily to be regarded as a simple filling of the LUMO orbital; rather, they are complex and bidirectional in many cases [46,47,48]. Also, local interactions between nitrogen atoms of FePc and the substrate may cause a change of the peak shape of N-K edge XAS spectra.

Further, the question arises whether or not the (neighbored) central metal atom is involved in the interaction at the interface. In Fe 2p XPS spectra (Figure 4a), we do not observe additional interface peaks, which would point to a chemical reaction or charge transfer to the Fe ion of the FePc molecule. Such interface peaks were found for reactive substrates like silver at about 1.5 eV lower BE compared to the multiplet signal of the bulklike multilayer film [25,49]. Also, an additional component at 707.0 eV (solid line in Figure 4a), as observed on the weaker interacting gold surfaces [50], seems to be absent. Therefore, Fe 2p photoemission spectra do not hint to an interaction between FePc and rutile TiO_2_(100) involving the Fe ion. The result reminds to FePc on TiO_x_ epitaxially grown on platinum(111) [27], although the detailed structure of substrate surfaces in both cases is different.

**Figure 9:**
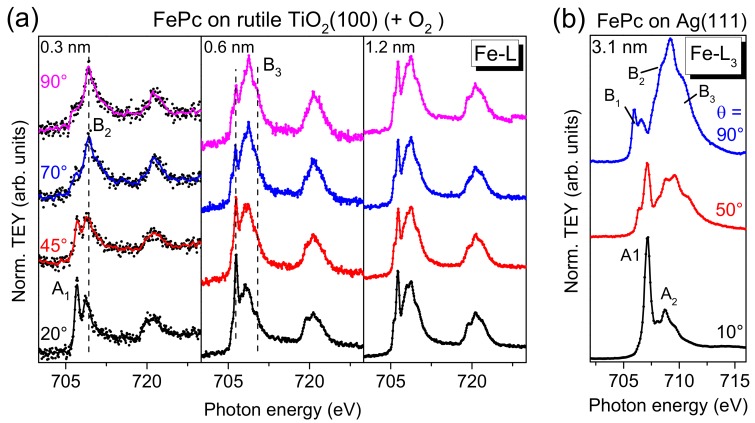
(**a**) Fe-L edge XAS spectra for FePc films of different thicknesses (0.3, 0.6, and 1.2 nm) on rutile TiO_2_(100). The substrate was annealed in the presence of oxygen during the last annealing step. The absence of additional features for low coverages indicates a comparably weak interaction. (**b**) The data are compared to a bulk-like FePc film (thickness 3.1 nm) on Ag(111) [13].

On the other hand, for TMPcs, the metal L-edge XAS spectra are very sensitive to the electronic structure, and thus to changes of the electronic configuration at the interface, in particular if the molecular orientation is known [13]. Angle-dependent Fe L_3,2_-edge XAS spectra for different FePc coverages on rutile TiO_2_(100) are shown in Figure 9a. We focus the discussion on the L_3_-edge at photon energies < 715 eV. For comparison, Fe L_3_-edge spectra of highly ordered, flat lying FePc molecules are shown in Figure 9b (3.1 nm FePc on Ag(111), data taken from [13]). Due to the flat lying adsorption geometry of FePc on Ag(111), at θ = 90° (normal incidence), transitions in the molecular plane are probed (denoted B1-B3), e.g., into d_xy_ and $d_{x^{2}-y^{2}}$ orbitals. In contrast, at θ = 10° transitions into orbitals with out-of-plane (*z*) contributions are observed (denoted A_1_ and A_2_).

In Figure 9a, a clear angular dependence of the spectral shape is visible in the spectra for low FePc coverages (0.3 and 0.6 nm) on rutile TiO_2_(100). The missing anisotropy of the spectra for the 1.2 nm film is due to an absence of a preferred molecular orientation at this film thickness (cf. discussion of N-K XAS spectra). In agreement with the degree of orientation obtained from N-K XAS spectra, the anisotropy is stronger for 0.3 nm compared to 0.6 nm. Most important, the monolayer spectra does not exhibit additional or shifted features as observed for strongly interacting systems, such as FePc on Ag(111) [25] or FePc on MnO [27], in good agreement with the discussion of Fe 2p PES spectra above. Such strong interactions can include a hybridization of Fe and substrate-related states and a redistribution of electrons at the Fe atom [13,25,27]. However, the feature denoted “A_1_” for the 0.3 nm spectrum at grazing incidence seems to exhibit a distinctly lower relative intensity compared to the reference spectra (FePc on Ag(111)), which cannot be understood alone by a slightly different molecular orientation (obtained from N-K XAS spectra) or by the different grazing incidence angle (20° and 10° with respect to the substrate surface in Figure 9a,b, respectively). This may point to a partial filling of the orbitals involved into these transitions due to weak interaction with the substrate or to a minor change of the electronic configuration of the Fe ion at the interface.

In summary, the absence of interface peaks in both XAS and XPS spectra points to a weak interaction at the studied FePc/rutile TiO_2_(100) interface; there is no evidence supporting a decomposition or oxidation of the molecule at the interface. The description of core-level spectra is essentially possible with the same model for coverages in the monolayer range and for thin films. The broadening of core-level spectra for low coverages can be understood by the adsorption at inhomogeneous adsorption sites, most likely accompanied by a complex partial charge transfer at the interface. A possible charge transfer involving N and Fe is further supported by changes of the shape of XAS spectra as a function of thickness. However, the weak interface peak in N 1s core-level spectra cannot be understood by such partial charge transfer processes into molecular orbitals. We assume that for few FePc molecules in the first monolayer, an additional, local interaction between nitrogen atoms and the rutile TiO_2_(100) substrate occurs.

### 2.3. FePc on Rutile TiO_2_(110)

As initially discussed, it might be expected that the face of the rutile crystal ((100) or (110)) distinctly affects the interaction at the interface to organic molecules. In other words, a possible reason for the strong interaction observed for FePc on rutile TiO_2_(110) [11] might be a more reactive (110) surface. Therefore, we studied in addition FePc on rutile TiO_2_(110). As for rutile TiO_2_(100), the last step in the preparation of the substrate (annealing) was performed either in the presence or absence of an oxygen partial pressure. Typically, we observe that the number of defects estimated from Ti 2p core-level spectra for the preparation in absence of oxygen is lower compared to rutile TiO_2_(100) (cf. Figure 2). The corresponding LEED image is shown in Figure S2b.

**Figure 10:**
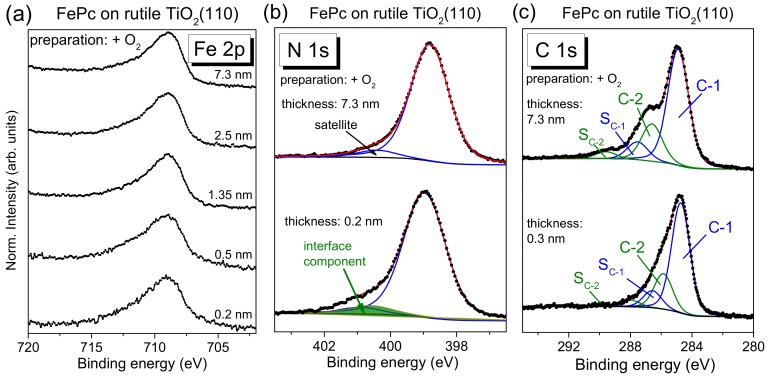
Thickness-dependent core-level spectra of FePc on rutile TiO_2_(110) (hυ = 1486.6 eV): (**a**) Fe 2p, (**b**) N 1s, (**c**) C 1s. The rutile crystal was prepared in presence of oxygen during the last annealing step. Similar to FePc on rutile TiO_2_(100), the spectra are essentially broadened for low coverages and a weak interface component is found in N 1s spectra only.

We discuss in Figure 10 core-level spectra of FePc on rutile TiO_2_(110), representative for the substrate preparation in the presence of oxygen during the last annealing step, and the corresponding fitting parameters are summarized in Table S6. In addition, spectra for the substrate preparation in absence of oxygen are shown as Supplementary Materials (Figure S5 and Table S7). As in the case of rutile TiO_2_(100), the presence of oxygen during the last annealing step of the substrate preparation affects only slightly the interface properties, and the corresponding core-level spectra can be described by the same model.

All core-level spectra of the bulk-like, thicker films show the expected shape, discussed in Section 2.2. Thickness-dependent changes of the peak shape of Fe 2p spectra are hardly visible. Similar to FePc on rutile TiO_2_(100), the C 1s spectrum for the sub-monolayer coverage (0.2 nm) in Figure 10 can be described by a Gaussian broadening of all components compared to the thick film (7.3 nm: 0.98 eV; 0.2 nm: 1.26 eV) and a slightly smaller C-1 to C-2 distance (1.1 eV). Also, the N 1s spectra can be described analogously to FePc on rutile TiO_2_(100): At low coverages, the main component is slightly broadened and additional intensity at a binding energy of ~401 eV is visible. The peak position of the main components of Fe 2p (708.9 eV), N 1s (399.0 eV), and C 1s (284.5 eV) spectra is almost independent of the film thickness (ΔBE ≤ 0.2 eV).

In summary, the development of core-level spectra as a function of the FePc thickness is very similar for FePc on TiO_2_(100) and TiO_2_(110), indicating very similar interface properties. Thus, the crystal face of the rutile cannot be the origin for the reported strong interaction of FePc on rutile TiO_2_(110) [11]. Rather, we hypothesize that the detailed preparation conditions, including the previous treatment of the crystal, may affect the number of surface defects and thus the interaction strength to the organic layer at the interface (cf. also Section 2.5).

### 2.4. FePcF_16_ on Rutile TiO_2_(100)

The fluorination of phthalocyanines mainly increases the ionization potential, whereas other electronic properties, such as the optical gap, are less affected, as previously shown, e.g., for CuPc and CoPc [47,51]. From UPS, we determine an ionization potential of 6.3 eV and 5.2 eV for perfluorinated FePcF_16_ and FePc, respectively. On the other hand, the optical band gap is very similar: From the onset of UV-vis spectra, we determine 1.58 (FePcF_16_) and 1.60 eV (FePc). Data are shown as Supplementary Materials (Figure S6). The increased ionization potential of fluorinated phthalocyanines may support an electron transfer to the molecule at interfaces, as shown for CoPc and CoPcF_16_ on Cu-intercalated graphene/Ni(111) [17]. Therefore, we studied interface properties at the example of FePcF_16_/rutile TiO_2_(100). The rutile was annealed in the presence of oxygen during all preparation steps.

**Figure 11:**
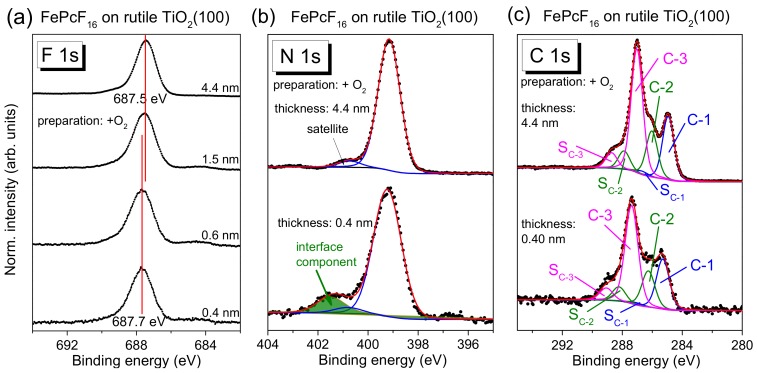
Thickness-dependent core-level spectra (excited with monochromatized Al Kα radiation, hυ = 1486.6 eV) of FePcF_16_ on rutile TiO_2_(100): (**a**) F 1s, (**b**) N 1s, (**c**) C 1s. The rutile crystal was prepared in the presence of oxygen during the last annealing step. The F 1s spectra shift by 0.2 eV to higher binding energy with decreasing film thickness. Similar to FePc on rutile TiO_2_(100) and TiO_2_(110), the spectra are essentially broadened for low coverages, and a weak interface component is found in N 1s spectra.

FePcF_16_-related F 1s, N 1s, and C 1s core-level spectra are shown in Figure 11, and the corresponding Fe 2p spectra are given in Figure S7. The F 1s spectra in Figure 11a exhibit one single peak, as expected from the chemical structure (Figure 1c). The spectral shape of the C 1s core-level spectra differs from C 1s of FePc due to the additional C–F bonds, visible as components at higher BE and a corresponding satellite, denoted C-3 and S_C-3_ in Figure 11c. The peak fit is in good agreement to C 1s spectra of related fluorinated phthalocyanines [17,52].

Most important, analogously to FePc on rutile TiO_2_(100), the spectral shape of all core-level spectra is almost independent of the film thickness. Small energetic shifts of 0.1–0.2 eV towards lower BE can be detected for all core levels with increasing film thickness (for Fe 2p, Figure S7). Many reasons can provoke such small energetic shifts. For the related fluorinated CuPcF_16_ on Au (a metal with a work function comparable to rutile), a pinning of the Fermi level at the interface close to the LUMO position for the “n-type” organic semiconductor was discussed, accompanied by band-bending like effects (the potential gradient in the organic film) [52].

The detailed analysis of N 1s and C 1s core-level spectra of FePcF_16_ by a peak fitting routine confirms that the same model can be applied for each film thickness, allowing a larger broadening for all components at lower film thickness (fitting parameters: Table S8). In the F 1s spectra, for low coverages, very weak intensity can be detected at lower binding energies (685 eV), which might indicate the breaking of some C–F bonds (cf. Section 2.5). In general, the development of the core-level spectra is very similar to the discussed FePc/rutile interfaces. Analogously to these interfaces, additional intensity is visible in the N 1s spectra at higher BE for low coverages (see green component and arrow in Figure 11b), discussed as a possible local interaction. We note that the interface component in N1s spectra is found at higher binding energy compared to FePc, and also the binding energy relative to the main component ΔBE is increased (ΔBE(FePcF_16_) = +2.3 eV, ΔBE(FePc) = +2.0 eV). The dependence of the binding energy on the kind of the molecule confirms that the molecules themselves are involved in the local interaction, rather than a scenario where a new nitrogen-containing compound is formed on the rutile surface.

In summary, the interaction of FePcF_16_ and rutile TiO_2_(100) is weak and very similar to the discussed FePc/rutile interfaces. Apparently, the fluorination of FePc does not affect significantly the interface properties to rutile TiO_2_(100).

### 2.5. FePcF_16_ on Defect-Rich, Hexagonal Rutile TiO_2_(100)

Finally, we investigate if a significantly higher number of defects on the TiO_2_ surface affects the interface properties distinctly. We have already shown that the exposure to oxygen during the last annealing step in the preparation of the rutile single crystal have only minor effects on interface properties of the deposited organic molecules.

**Figure 12:**
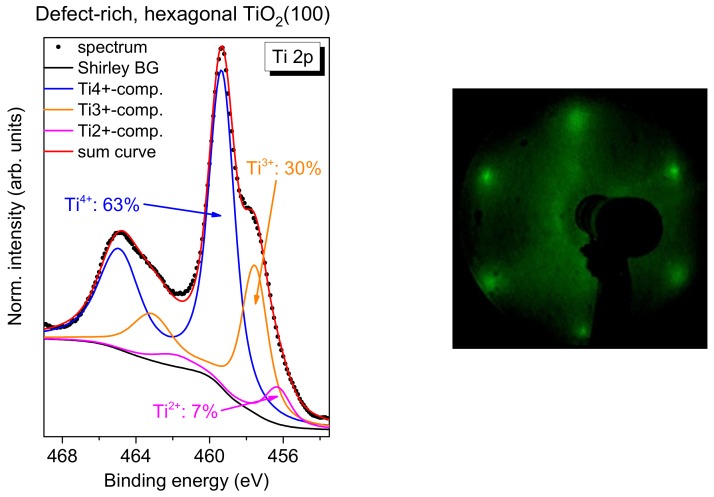
Left: Ti 2p spectrum of a defect-rich rutile TiO_2_(100) (excited with monochromatized Al K α radiation, hυ = 1486.6 eV), prepared in absence of oxygen (3–4 annealing steps). Right: Hexagonal LEED pattern of an incomplete layer TiO_2_(100) (E = 60 eV).

However, if rutile TiO_2_(100) is prepared without (or with significantly less) exposure to oxygen during all annealing steps, a more defect-rich substrate can be prepared. For TiO_2_(110), rosette-like network patches are observed; they can be understood by an incomplete 1 × 1 structure [53,54]. The annealing of the rutile TiO_2_(100) single crystal in the absence of oxygen (3-4 annealing steps) results in a hexagonal surface structure similar to the reported structures on TiO_2_(110) [53,54]. A typical Ti 2p spectrum, together with an LEED image, is shown in Figure 12. In contrast to the systems discussed before, the Ti 2p spectrum cannot be explained on the sole basis of a high number of Ti^3+^ defects, but rather an additional component for Ti^2+^ has to be introduced (fitting parameters: Table S9).

**Figure 13:**
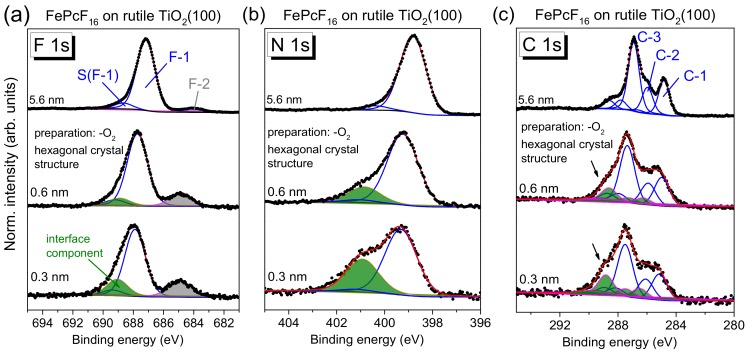
Thickness-dependent core-level spectra of FePcF_16_ on defect-rich rutile TiO_2_(100) (excited with monochromatized Al K α radiation, hυ = 1486.6 eV): (**a**) F 1s, (**b**) N 1s, (**c**) C 1s. The rutile crystal was prepared in absence of oxygen during several annealing steps. In contrast to FePcF_16_ on rutile TiO_2_(100) prepared in presence of oxygen during all annealing steps, distinct interface components are observed.

The core-level spectra of FePcF_16_ in Figure 13 (F 1s (a), N 1s (b), and C 1s (c)) show distinct changes with film thickness—in contrast to FePcF_16_ on rutile TiO_2_(100) prepared in the presence of oxygen (Figure 11). The corresponding Fe 2p spectra are shown in Figure S8. The broad multiplet structures of the Fe 2p spectra, which may depend also on the (thickness-dependent) arrangement of the FePcF_16_ molecules in thin films [55,56], hamper, however, a more detailed analysis of these spectra.

Compared to FePcF_16_ on rutile TiO_2_(100) prepared in the presence of oxygen (Figure 11), the N 1s spectra in Figure 13 show distinctly more intense interface components (green peaks in Figure 13b) for low coverages at a binding energy of 401.0 eV, indicating that more molecules undergo a strong interaction at this interface. The relative intensity is 16.0% and of 31.4% for 0.6 and 0.3 nm thick films, respectively. For the N 1s peak fit, a thickness independent of N 1s satellite intensity was assumed. All fit parameters of spectra shown in Figure 13 are summarized in Tables S10–S14.

Analogously to N 1s, the peak fit of F 1s spectra reveals an interface component at higher binding energy with respect to the main peak (green component in Figure 13a). In addition, an interface component is visible at about 685 eV in Figure 13a (gray). The binding energy of this component in the range of Ti-F surface fluorides [57,58]. Therefore, the presence of this component may indicate the breaking of some C–F bonds of FePcF_16_ at the interface and the formation of new bonds with the TiO_2_ substrate. As a consequence, a covalent bond between the phthalocyanine and the substrate or a new C–H bond might be formed.

Also, changes of the shape of C 1s spectra with decreasing film thickness are clearly visible in Figure 13. Whereas in the thicker film (5.6 nm), the three components (labeled C-1, C-2, and C-3) and the corresponding satellites (cf. Figure 11) are well resolved, at lower coverages, additional intensity appears at higher BE (see arrows in Figure 13c). However, in contrast to N 1s spectra, the introduction of a single interface component accompanied by a broadening of all components is not sufficient to describe the spectra. Therefore, we applied a similar model, as reported for FePc on (reactive) TiO_2_(110) [11]. In this model, the C 1s signal has contributions from two types of FePc, assigned to first- and second-layer molecules [11]. In a similar manner, we describe the C 1s spectra of the 0.3 and 0.6 nm coverages in Figure 13c by two sets of spectra, the first with a binding energy similar to the 5.6 nm thick film and the second with a binding energy shifted to higher values (green peaks). Both the binding energy of all components relative to the main component and their relative intensities were kept constant for both sets of spectra (fitting parameters summarized in Tables S11–S13). Compared to the spectra of thicker films in Figure 11c and Figure 13c, the relative energies of the components are slightly different, which might be due to a different charge distribution for molecules directly at the interface. The total relative intensity of the green (energetically shifted) components in Figure 13c is 25% and 17% for the coverages of 0.3 and 0.6 nm, respectively. The energy shift is 1.2–1.3 eV with respect to the main spectrum (blue curves in Figure 13c). Thus, the C 1s signal for lower coverages can be described by contributions from two types of FePcF_16_ molecules: Type I is almost unaffected at the interface, whereas the spectrum of type II is shifted towards higher binding energies.

The origin of such energetic shifts in C 1s spectra to higher binding energy could be (1) a chemical shift due to oxidation of the corresponding atom(s) or (2) a shift of the energy levels of the organic semiconductor relative to the reference (Fermi) level of the spectrometer due to a different energy level alignment at the interface to the substrate. Since a chemical shift to more than 1 eV higher binding energy would imply an oxidation of the corresponding species by about one oxidation number, scenario (1) seems to be unlikely. Also, the binding energies of all carbon species of type II FePcF_16_ molecules are shifted by similar values, pointing to a shift of the reference level (scenario (2)). The different energy level alignment for type II FePcF_16_ molecules might be caused by a distinct (integer) charge transfer from the substrate to the molecules, accompanied by the occupation of formerly unoccupied orbitals (i.e., the LUMO) of the molecule. This might also explain the presence of the intense interface component in N 1s and F 1s spectra (green components in Figure 13a,b) at 1.6 eV and 1.15 eV higher binding energy with respect to the main components, respectively. These interface peaks might contain contributions of type II FePcF_16_ molecules, shifted to higher binding energy. The fact that the green components in Figure 13a–c do not dominate the spectra demonstrates that not all molecules are involved in the stronger interaction. Further, the essentially similar C 1s peak shape of type I and type II FePcF_16_ molecules indicates that the chemical structure of the molecule at the reactive sites is almost the same as on non-reactive sites, even if some of the C–F bonds are broken (cf. Figure 13a).

## 3. Materials and Methods

The rutile single crystals were purchased from Pi-KEM Ltd., England. Single crystals were cleaned by sputtering and annealing cycles and checked for contaminants. If not otherwise mentioned, the maximum sputter voltage was 0.9 kV at an Argon (Westfalen AG, 99.999%, Münster, Germany) pressure of 5 × 10^−5^ mbar. The annealing temperature was between 840 and 870 K. The initial annealing cycles were carried out in an oxygen atmosphere of 1 × 10^−6^ mbar O_2_. The final annealing cycle was carried out with and without O_2_ to test the influence of the oxygen on the interface properties. FePc and FePcF_16_ powders were purchased from Sigma Aldrich Chemie GmbH (dye content 90%, Steinheim, Germany) and Synthon Chemicals GmbH & Co. KG (Bitterfeld-Wolfen, Germany), respectively. The powders were resublimed prior to application.

X-ray absorption spectroscopy (XAS) and photoemission (PES) measurements using synchrotron radiation were performed at BESSY II (Helmholtz-Zentrum, Berlin, Germany) at the LowDosePES endstation of the PM4 beamline [59]. Polarization-dependent X-ray absorption spectra at the N-K, Fe-L, and Ti-L edges were acquired at different angles of the incident p-polarized light with respect to the surface plane (with θ = 90° normal incidence and 20° grazing incidence) in total-electron yield (TEY, drain current) mode. The energy resolution was set to 100 meV at photon energy 400 eV. The Ti 2p XPS and the Ti-L XAS spectrum of Figure 3c were measured at KARA (Karlsruhe, Germany).

PES measurements in the home-lab were performed in ultra-high vacuum systems equipped with a twin anode standard sources (Al and Mg Kα), a monochromatized standard source (Al Kα), PHOIBOS 100 or 150 hemispherical analyzers (SPECS), and a four-stage LEED (SpectaLEED Omicron, Germany). The calibration of the binding energy scale is referred to the signal positions of Cu 2p_3/2_ (932.6 eV), Ag 3d_5/2_ (368.2 eV), and Au 4f_7/2_ (84.0 eV).

The nominal layer thicknesses were estimated from XPS intensity ratios using photoemission cross sections from Yeh and Lindau [60]. The monolayer (ML) thickness was assumed to be 0.34 nm for lying molecules, considering the molecule–molecule distance from powder diffraction data of TMPc α-polymorph [61,62,63]. Peak fitting of XPS and XAS spectra was performed using the programs Unifit 2015 and Unifit 2018 [64]. The program VESTA was used to illustrate the crystal structures in Figure 1 [65].

## 4. Conclusions

In summary, interface properties of FePc on rutile TiO_2_(100) and TiO_2_(110) surfaces were compared. FePcF_16_ was studied on rutile TiO_2_(100) and on a defect-rich, hexagonal rutile TiO_2_(100) substrate. Weak interactions were observed for all studied interfaces between FePc or FePcF_16_ and rutile, as long as the substrate was exposed to oxygen during the annealing steps of the preparation procedure. The absence of oxygen in the last annealing step only had only minor influence on interface properties. The shape of XPS core-level spectra was almost independent of the film sites, most likely accompanied by a complex partial charge transfer at the interface. The occurrence of a weak interface peak in N 1s core-level spectra point to a local interaction between few nitrogen atoms in the first monolayer and substrate. In contrast, the absence of oxygen during several steps of the substrate preparation procedure resulted in a more reactive, defect-rich substrate surface. The XPS core-level spectra of FePcF_16_ on such a defect-rich, hexagonal rutile TiO_2_(100) surface can be essentially described by two types of molecules with different energy level alignment, possibly caused by an occupation of the LUMO of some molecules at the interface. Although the chemical structure of the molecule at the reactive sites was almost preserved, F 1s spectra indicate that some of the C–F bonds were broken. Thus, we conclude that the detailed preparation conditions affect the interaction strength at the interfaces considerably.

## Supplementary Materials

The following are available online at https://www.mdpi.com/1420-3049/24/24/4579/s1: Figure S1. Ti 2p spectra of rutile TiO_2_(100) prepared in an oxygen atmosphere; Figure S2. Typical LEED 1 × 1 pattern of (**a**) rutile TiO_2_(100) and (**b**) rutile TiO_2_(110); Figure S3. Peak fit of N 1s (**a**) and C 1s (**b**) core-level spectra of 4.2 and 0.3 nm thick FePc films on rutile TiO_2_(100); Figure S4. N-K Edge X-ray absorption spectra for a thick FePc film on rutile TiO_2_(110); Figure S5. Thickness-dependent core-level spectra of FePc on rutile TiO_2_(110); Figure S6. UV-vis spectra of FePc (black) and FePcF_16_ (red) evaporated on glass; Figure S7. Thickness-dependent Fe 2p core-level spectra of FePcF_16_ on rutile TiO_2_(100); Figure S8. Thickness-dependent Fe 2p core-level spectra of FePcF_16_ on defect-rich rutile TiO_2_(100); Tables S1–S14. Peak fit parameters for spectra shown in the paper.

## References

[1] Diebold U. (2003). The surface science of titanium dioxide. Surf. Sci. Rep..

[2] O’regan B., Grätzel M. (1991). A low-cost, high-efficiency solar cell based on dye-sensitized colloidal TiO_2_ films. Nature.

[3] Grätzel M. (2000). Perspectives for dye-sensitized nanocrystalline solar cells. Prog. Photovolt. Res. Appl..

[4] Grätzel M. (2001). Photoelectrochemical cells. Nature.

[5] Sharma G.D., Kumar R., Roy M.S. (2006). Investigation of charge transport, photo generated electron transfer and photovoltaic response of iron phthalocyanine (FePc): TiO_2_ thin films. Sol. Energy Mater. Sol. Cells.

[6] Urbani M., Ragoussi M.-E., Nazeeruddin M.K., Torres T. (2019). Phthalocyanines for dye-sensitized solar cells. Coord. Chem. Rev..

[7] Li L., Yan J., Wang T., Zhao Z.-J., Zhang J., Gong J., Guan N. (2015). Sub-10 nm rutile titanium dioxide nanoparticles for efficient visible-light-driven photocatalytic hydrogen production. Nat. Commun..

[8] Wang D., Leng Z., Hüben M., Oeser M., Steinauer B. (2016). Photocatalytic pavements with epoxy-bonded TiO_2_-containing spreading material. Constr. Build. Mater..

[9] Chen X., Mao S.S. (2007). Titanium Dioxide Nanomaterials: Synthesis, Properties, Modifications, and Applications. Chem. Rev..

[10] Ishida N., Fujita D. (2012). Adsorption of co-phthalocyanine on the rutile TiO_2_ (110) surface: A scanning tunneling microscopy/spectroscopy study. J. Phys. Chem. C.

[11] Palmgren P., Nilson K., Yu S., Hennies F., Angot T., Layet J.M., Le Lay G., Göthelid M. (2008). Strong Interactions in Dye-Sensitized Interfaces. J. Phys. Chem. C.

[12] Sinha S., Islam A.M., Vorokhta M., Mukherjee M. (2017). Interaction at the F16CuPc/TiO_2_ interface: A photoemission and X-ray absorption study. J. Phys. Chem. C.

[13] Peisert H., Uihlein J., Petraki F., Chassé T. (2015). Charge transfer between transition metal phthalocyanines and metal substrates: The role of the transition metal. J. Electron. Spectrosc. Relat. Phenom..

[14] Henderson M.A. (1994). The influence of oxide surface structure on adsorbate chemistry: Desorption of water from the smooth, the microfaceted and the ion sputtered surfaces of TiO_2_ (100). Surf. Sci..

[15] Henderson M.A. (1996). Structural sensitivity in the dissociation of water on TiO_2_ single-crystal surfaces. Langmuir.

[16] Bourgeois S., Jomard F., Perdereau M. (1992). Use of isotopic labelling in a SIMS study of the hydroxylation of TiO_2_ (100) surfaces. Surf. Sci..

[17] Balle D., Adler H., Grüninger P., Karstens R., Ovsyannikov R., Giangrisostomi E., Chassé T., Peisert H. (2017). Influence of the Fluorination of CoPc on the Interfacial Electronic Structure of the Coordinated Metal Ion. J. Phys. Chem. C.

[18] Nowotny M.K., Sheppard L.R., Bak T., Nowotny J. (2008). Defect chemistry of titanium dioxide. application of defect engineering in processing of TiO_2_-based photocatalysts. J. Phys. Chem. C.

[19] Nolan M., Elliott S.D., Mulley J.S., Bennett R.A., Basham M., Mulheran P. (2008). Electronic structure of point defects in controlled self-doping of the TiO_2_ (110) surface: Combined photoemission spectroscopy and density functional theory study. Phys. Rev. B.

[20] Wang L.-Q., Baer D.R., Engelhard M.H., Shultz A.N. (1995). The adsorption of liquid and vapor water on TiO_2_(110) surfaces: The role of defects. Surf. Sci..

[21] Pouilleau J., Devilliers D., Groult H., Marcus P. (1997). Surface study of a titanium-based ceramic electrode material by X-ray photoelectron spectroscopy. J. Mater. Sci..

[22] Wang L.Q., Baer D.R., Engelhard M.H. (1994). Creation of variable concentrations of defects on TiO_2_ (110) using low-density electron beams. Surf. Sci..

[23] Casu M.B., Braun W., Bauchspiess K.R., Kera S., Megner B., Heske C., Thull R., Umbach E. (2008). A multi-technique investigation of TiO_2_ films prepared by magnetron sputtering. Surf. Sci..

[24] Kucheyev S., Van Buuren T., Baumann T., Satcher J., Willey T., Meulenberg R., Felter T., Poco J., Gammon S., Terminello L. (2004). Electronic structure of titania aerogels from soft x-ray absorption spectroscopy. Phys. Rev. B.

[25] Petraki F., Peisert H., Aygul U., Latteyer F., Uihlein J., Vollmer A., Chassé T. (2012). Electronic structure of FePc and interface properties on Ag (111) and Au (100). J. Phys. Chem. C.

[26] Åhlund J., Nilson K., Schiessling J., Kjeldgaard L., Berner S., Mårtensson N., Puglia C., Brena B., Nyberg M., Luo Y. (2006). The electronic structure of iron phthalocyanine probed by photoelectron and x-ray absorption spectroscopies and density functional theory calculations. J. Chem. Phys..

[27] Glaser M., Peisert H., Adler H., Polek M., Uihlein J., Nagel P., Merz M., Schuppler S., Chassé T. (2015). Transition-Metal Phthalocyanines on Transition-Metal Oxides: Iron and Cobalt Phthalocyanine on Epitaxial MnO and TiO x Films. J. Phys. Chem. C.

[28] Peisert H., Knupfer M., Fink J. (2002). Electronic structure of partially fluorinated copper phthalocyanine (CuPCF4) and its interface to Au (100). Surf. Sci..

[29] Brena B., Luo Y., Nyberg M., Carniato S., Nilson K., Alfredsson Y., Åhlund J., Mårtensson N., Siegbahn H., Puglia C. (2004). Equivalent core-hole time-dependent density functional theory calculations of carbon 1 s shake-up states of phthalocyanine. Phys. Rev. B.

[30] Peisert H., Knupfer M., Fink J. (2003). Comparison of the electronic structure of CuPCF4/ITO and CuPCF4/Au interfaces. Synth. Met..

[31] Schöll A., Zou Y., Jung M., Schmidt T., Fink R., Umbach E. (2004). Line shapes and satellites in high-resolution x-ray photoelectron spectra of large pi-conjugated organic molecules. J. Chem. Phys..

[32] Ishii H., Sugiyama K., Ito E., Seki K. (1999). Energy level alignment and interfacial electronic structures at organic metal and organic organic interfaces. Adv. Mater..

[33] Gruninger P., Polek M., Ivanovic M., Balle D., Karstens R., Nagel P., Merz M., Schuppler S., Ovsyannikov R., Bettinger H.F. (2018). Electronic structure of hexacene and interface properties on Au (110). J. Phys. Chem. C.

[34] Khoshkhoo M.S., Peisert H., Chasse T., Scheele M. (2017). The role of the density of interface states in interfacial energy level alignment of PTCDA. Org. Electron..

[35] Hwang J., Wan A., Kahn A. (2009). Energetics of metal-organic interfaces: New experiments and assessment of the field. Mater. Sci. Eng. R-Rep..

[36] Oehzelt M., Koch N., Heimel G. (2014). Organic semiconductor density of states controls the energy level alignment at electrode interfaces. Nat. Commun..

[37] Peisert H., Petershans A., Chasse T. (2008). Charge transfer and polarization screening at organic/metal interfaces: Distinguishing between the first layer and thin films. J. Phys. Chem. C.

[38] Peisert H., Biswas I., Knupfer M., Chasse T. (2009). Orientation and electronic properties of phthalocyanines on polycrystalline substrates. Phys. Status Solidi B.

[39] Biswas I., Peisert H., Casu M.B., Schuster B.E., Nagel P., Merzz M., Schuppler S., Chasse T. (2009). Initial molecular orientation of phthalocyanines on oxide substrates. Phys. Status Solidi A.

[40] Willey T.M., Bagge-Hansen M., Lee J.R.I., Call R., Landt L., van Buuren T., Colesniuc C., Monton C., Valmianski I., Schuller I.K. (2013). Electronic structure differences between H_2-_, Fe-, Co-, and Cu-phthalocyanine highly oriented thin films observed using NEXAFS spectroscopy. J. Chem. Phys..

[41] Aristov V.Y., Molodtsova O.V., Maslyuk V.V., Vyalikh D.V., Bredow T., Mertig I., Preobrajenski A.B., Knupfer M. (2010). Electronic properties of potassium-doped FePc. Org. Electron..

[42] Uihlein J., Polek M., Glaser M., Adler H., Ovsyannikov R., Bauer M., Ivanovic M., Preobrajenski A.B., Generalov A.V., Chassé T. (2015). Influence of graphene on charge transfer between CoPc and metals: The role of graphene–substrate coupling. J. Phys. Chem. C.

[43] Glaser M. (2016). Untersuchung der elektronischen Wechselwirkungen an Grenzflächen zwischen Organischen Halbleitermaterialien und ultra-dünnen Oxidfilmen. Ph.D. Thesis.

[44] Rocco M.L.M., Frank K.H., Yannoulis P., Koch E.E. (1990). Unoccupied electronic structure of phthalocyanine films. J. Chem. Phys..

[45] Floreano L., Cossaro A., Gotter R., Verdini A., Bavdek G., Evangelista F., Ruocco A., Morgante A., Cvetko D. (2008). Periodic arrays of Cu-phthalocyanine chains on Au (110). J. Phys. Chem. C.

[46] Petraki F., Peisert H., Uihlein J., Aygül U., Chassé T. (2014). CoPc and CoPcF16 on gold: Site-Specific charge-transfer processes. Beilstein J. Nanotechnol..

[47] Lindner S., Treske U., Knupfer M. (2013). The complex nature of phthalocyanine/gold interfaces. Appl. Surf. Sci..

[48] Huang Y., Wruss E., Egger D., Kera S., Ueno N., Saidi W., Bucko T., Wee A., Zojer E. (2014). Understanding the adsorption of CuPc and ZnPc on noble metal surfaces by combining quantum-mechanical modelling and photoelectron spectroscopy. Molecules.

[49] Bai Y., Buchner F., Wendahl M.T., Kellner I., Bayer A., Steinrück H.-P., Marbach H., Gottfried J.M. (2008). Direct metalation of a phthalocyanine monolayer on Ag (111) with coadsorbed Iron atoms. J. Phys. Chem. C.

[50] Schmid M., Zirzlmeier J., Steinruck H.P., Gottfried J.M. (2011). Interfacial interactions of Iron(II) tetrapyrrole complexes on Au (111). J. Phys. Chem. C.

[51] Peisert H., Knupfer M., Fink J. (2002). Energy level alignment at organic/metal interfaces: Dipole and ionization potential. Appl. Phys. Lett..

[52] Peisert H., Knupfer M., Schwieger T., Fuentes G.G., Olligs D., Fink J., Schmidt T. (2003). Fluorination of copper phthalocyanines: Electronic structure and interface properties. J. Appl. Phys..

[53] Li M., Hebenstreit W., Gross L., Diebold U., Henderson M., Jennison D., Schultz P., Sears M. (1999). Oxygen-induced restructuring of the TiO_2_ (110) surface: A comprehensive study. Surf. Sci..

[54] Li M., Hebenstreit W., Diebold U., Tyryshkin A.M., Bowman M.K., Dunham G.G., Henderson M.A. (2000). The influence of the bulk reduction state on the surface structure and morphology of rutile TiO_2_ (110) single crystals. J. Phys. Chem. B.

[55] Belser A., Karstens R., Nagel P., Merz M., Schuppler S., Chassé T., Peisert H. (2018). Interaction Channels Between Perfluorinated Iron Phthalocyanine and Cu (111). Phys. Status Solidi (b).

[56] Belser A., Karstens R., Grüninger P., Nagel P., Merz M., Schuppler S., Suturina E.A., Chassé A., Chassé T., Peisert H. (2018). Spin state in perfluorinated FePc films on Cu(111) and Ag(111) in dependence on film thickness. J. Phys. Chem. C.

[57] Yu J.C., Yu J.G., Ho W.K., Jiang Z.T., Zhang L.Z. (2002). Effects of F- doping on the photocatalytic activity and microstructures of nanocrystalline TiO_2_ powders. Chem. Mater..

[58] Yu J.G., Xiang Q.J., Ran J.R., Mann S. (2010). One-Step hydrothermal fabrication and photocatalytic activity of surface-fluorinated TiO_2_ hollow microspheres and tabular anatase single micro-crystals with high-energy facets. Crystengcomm.

[59] Giangrisostomi E., Ovsyannikov R., Sorgenfrei F., Zhang T., Lindblad A., Sassa Y., Cappel U.B., Leitner T., Mitzner R., Svensson S. (2018). Low dose photoelectron spectroscopy at BESSY II: Electronic structure of matter in its native state. J. Electron. Spectrosc..

[60] Yeh J.J., Lindau I. (1985). Atomic subshell photoionization cross sections and asymmetry parameters: 1 ≤ Z ≤ 103. Atom. Data Nucl. Data Tables.

[61] Ballirano P., Caminiti R., Ercolani C., Maras A., Orrù M.A. (1998). X-ray powder diffraction structure reinvestigation of the α and β forms of Cobalt phthalocyanine and kinetics of the α → β phase transition. J. Am. Chem. Soc..

[62] Gould R.D. (1996). Structure and electrical conduction properties of phthalocyanine thin films. Coord. Chem. Rev..

[63] Evangelisti M., Bartolomé J., de Jongh L.J., Filoti G. (2002). Magnetic properties of iron(II) phthalocyanine. Phys. Rev. B.

[64] Hesse R., Chassé T., Streubel P., Szargan R. (2004). Error estimation in peak-shape analysis of XPS core-level spectra using UNIFIT 2003: How significant are the results of peak fits?. Surf. Interface Anal..

[65] Momma K., Izumi F. (2011). VESTA 3 for three-dimensional visualization of crystal, volumetric and morphology data. J. Appl. Crystallogr..

