# Supplementary material for: FePc and FePcF16 on Rutile TiO2(110) and (100): Influence of the Substrate Preparation on the Interaction Strength
Source: Molecules. 2019 Dec 13;24(24):4579. doi: 10.3390/molecules24244579 (PMC6943671; doi:10.3390/molecules24244579)
Supplement: Supplementary file 1 [file molecules-24-04579-s001.pdf]

Supplementary Materials: FePc and $FePcF_{16}$ on Rutile TiO_2(110) and (100): Influence of the Substrate Preparation on the Interaction Strength

Reimer Karstens,[1] Mathias Glaser,[1] Axel Belser,[1] David Balle,[1] Małgorzata Polek,[1] Ruslan Ovsyannikov,[2] Erika Giangrisostomi,[2] Thomas Chassé,[1,3] Heiko Peisert[1,*]

[1] Institute of Physical and Theoretical Chemistry, University of Tübingen, Auf der Morgenstelle 18, 72076 Tübingen, Germany
[2] Institute for Methods and Instrumentation in Synchrotron Radiation Research, Helmholtz-Zentrum Berlin für Materialien und Energie GmbH, Albert-Einstein-Strasse 15, 12489 Berlin, Germany
[3] Center for Light-Matter Interaction, Sensors & Analytics (LISA+) at the University of Tübingen, Auf der Morgenstelle 18, 72076 Tübingen, Germany
* Correspondence: heiko.peisert@uni-tuebingen.de

1. Peak fit parameters of Ti 2p spectrum (Figure 2(b))

Table S1: Peak fit parameters of rutile TiO_2(100) prepared without O_2 during the last annealing step. Measured using a standard X-ray source (excitation energy of 1486.6 eV). BE: binding energy, LW: Lorentzian width, GW: Gaussian width

FePc/TiO_2(100) without O_2	**D1 (Ti^{4+})**<br>Component1<br>Component2	**D2 (Ti^{3+})**<br>Component1<br>Component2
peak position<br>[BE, eV]	459.29<br>464.97	457.34<br>463.02
LW [eV]	1.30<br>1.94	1.30<br>1.94
GW [eV]	1.23<br>1.67	1.23<br>1.67
rel. area [%]	76	24

2. Surface sensitivity of Ti 2p spectra

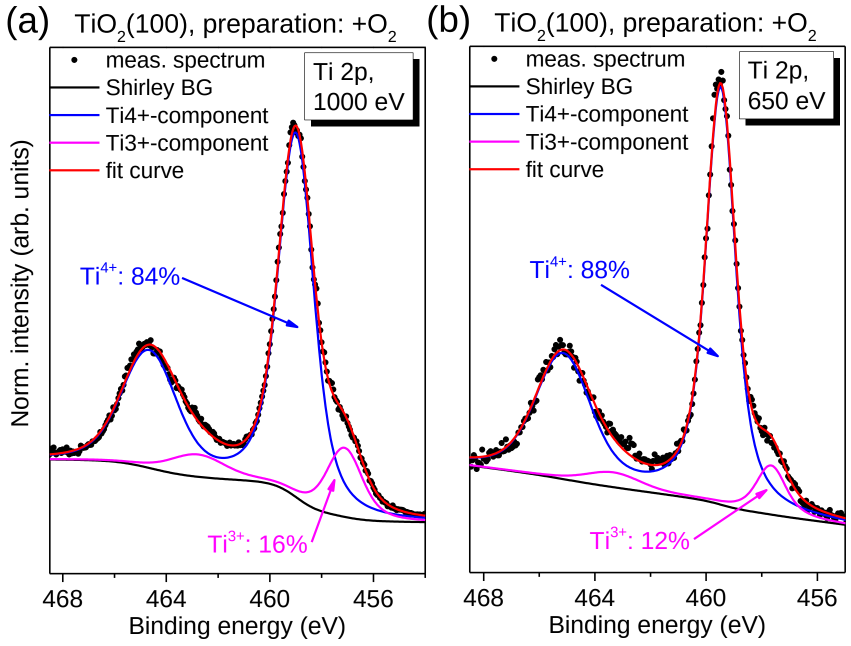

Figure S1: Ti 2p spectra of rutile $TiO_2(100)$ prepared in an oxygen atmosphere, which are measured with two different excitation energies. (a) 1000 eV, (b) 650 eV. The lower excitation energy is more surface-sensitive. Then, the change in the relative intensity of Ti^{3+} (4%) indicates that the defects are almost evenly distributed with a small preference for the bulk.

Table S2: Peak fit parameters of rutile $TiO_2(100)$ prepared in presence of O_2 measured with an excitation energy of 1000 eV (Figure S1(a)).

FePc/TiO_2(100) with O_2, E_{exc} = 1000 eV	**D1 (Ti^{4+})** Component1 Component2	**D2 (Ti^{3+})** Component1 Component2
peak position [BE, eV]	458.99<br>464.66	457.12<br>462.79
LW [eV]	0.78<br>0.79	0.78<br>0.79
GW [eV]	1.16<br>2.04	1.16<br>2.04
rel. area [%]	84	16

Table S3: Peak fit parameters of rutile $TiO_2(100)$ prepared in presence of O_2 measured with an excitation energy of 650 eV (Figure S1(a)).

FePc/$TiO_2(100)$ with O_2, E_{exc} = 650 eV	**D1 (Ti^{4+})** Component1 Component2	**D2 (Ti^{3+})** Component1 Component2
peak position [BE, eV]	459.47<br>465.14	457.71<br>463.38
LW [eV]	0.68<br>0.88	0.68<br>0.88
GW [eV]	0.82<br>1.76	0.82<br>1.76
rel. area [%]	88	12

3. Preparation of the substrates: LEED

In Figure S2(a) and S2(b) the typical 1 × 1 LEED pattern of rutile $TiO_2(100)$ and rutile $TiO_2(110)$ prepared with/without oxygen during the last annealing step are shown. Compared to Figure S2(a), the LEED pattern of Figure S2(c) is broadened and the background is increased, indicating the presence of smaller defect-free rutile $TiO_2(100)$ domains at the surface. Additional streaks along the (001) crystallographic axis in SI Figure S2(c) may indicate the presence of additional ordered structures on the substrate surface. An example of a LEED pattern for rutile $TiO_2(110)$ with a higher number of defects (prepared without oxygen in the last annealing step) is shown in Figure S2(d).

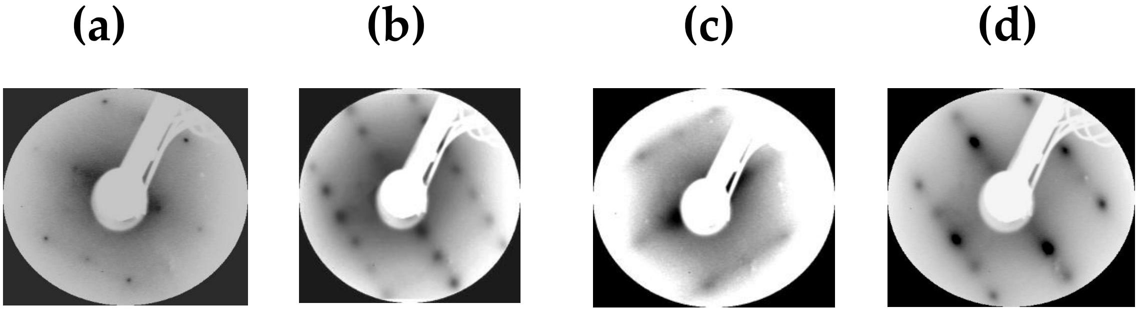

Figure S2: Typical LEED 1x1 pattern of (a) rutile $TiO_2(100)$ and (b) rutile $TiO_2(110)$. a) and b) were prepared with oxygen in the last step, while c) and d) were prepared without oxygen exposure during the last annealing step. The energy E of the low energetic electrons was about 90 eV. Also for a defect rich rutile $TiO_2(100)$ surface, the 1 × 1 pattern are detectable (c) (E = 80 eV), even if streaks and a stronger background appear, pointing to a smaller size of ordered domains. For a defect rich rutile $TiO_2(110)$ surface, the 1x1 pattern are also detectable (d) (E = 89 eV).

4. N 1s and C 1s peakfit parameters for FePc on rutile $TiO_2(100)$ prepared in presence of oxygen

Table S4: Peak fit parameters for spectra shown in Figure 5(a) of the manuscript. Top: N 1s (top). Bottom: C 1s.

**5.1 nm**	**S1**<br>N-1	**S2**<br>S(N-1)	**0.3 nm**	**S1**<br>N-1	**S2**<br>NE-1	**S3**<br>S(N-1)
peak position [BE, eV]	398.8	400.4		399.0	401.05	400.6
LW [eV]	0.46	0.46		0.46	0.46	0.46
GW [eV]	1.13	1.13		1.24	1.24	1.24
rel. area [%]	94	6		87	7	6

**5.1 nm**	**S1**<br>C-1	**S2**<br>S(C-1)	**S3**<br>C-2	**S4**<br>S(C-2)	**0.3 nm**	**S1**<br>C-1	**S2**<br>S(C-1)	**S3**<br>C-2	**S4**<br>S(C-2)
peak position [BE, eV]	284.4	286.2	285.6	287.7		284.6	286.3	285.7	287.9
LW [eV]	0.26	0.26	0.26	0.26		0.24	0.24	0.24	0.24
GW [eV]	1.02	1.02	1.02	1.02		1.26	1.26	1.26	1.26
rel. area [%]	65.0	10.1	20.9	4.0		65.0	10.1	20.9	4.0

5. N 1s and C 1s peakfits of FePc on rutile TiO_2(100) prepared in absence of oxygen

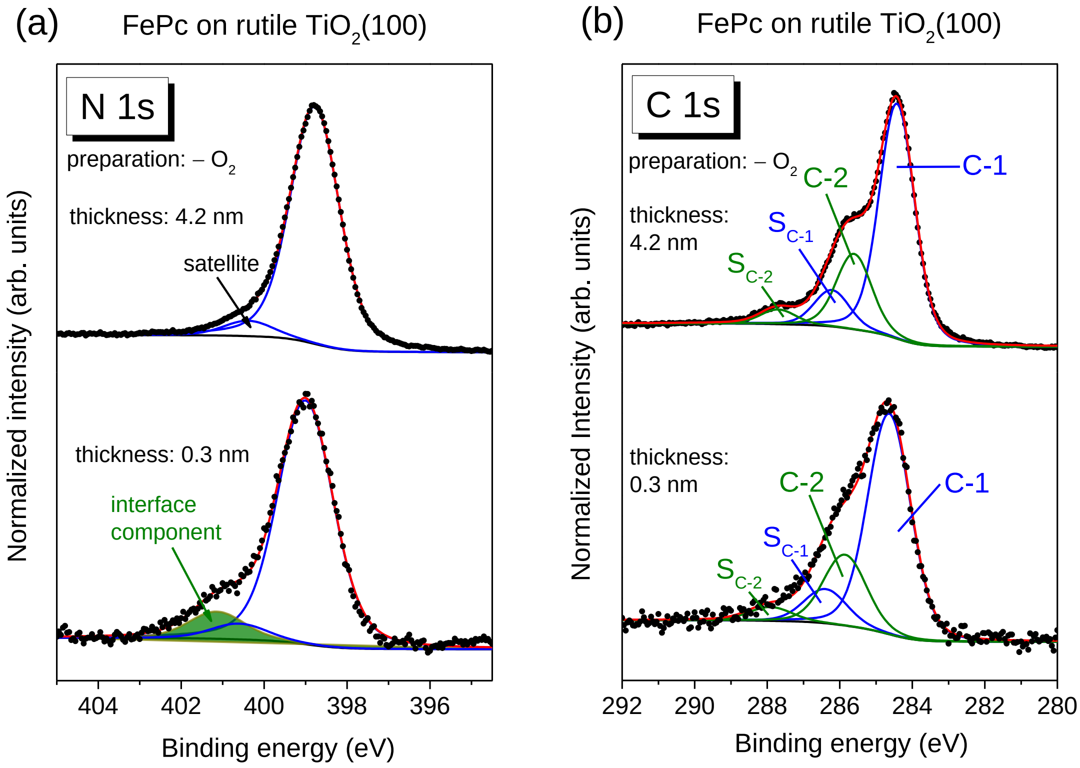

Figure S3: Peak fit of N 1s (a) and C 1s (b) core level spectra of 4.2 nm and 0.3 nm thick FePc films on rutile TiO_2(100) (hν = 1486.6 eV), prepared in absence of oxygen during the last annealing step.

Table S5: Peak fit parameters for peak fits shown in Figure S3. Top: N 1s (N-1: main component, S(N-1): satellite of N-1, NE-1: interface component). Bottom: C 1s (notation see main text).

**4.2 nm**	**S1** N-1	**S2** S(N-1)	**0.3 nm**	**S1** N-1	**S2** NE-1	**S3** S(N-1)
peak position [BE, eV]	398.8	400.4		399.0	401.1	400.6
LW [eV]	0.42	0.42		0.46	0.46	0.46
GW [eV]	1.16	1.16		1.33	1.33	1.33
rel. area [%]	94	6		85	9	6

**4.2 nm**	**S1** C-1	**S2** S(C-1)	**S3** C-2	**S4** S(C-2)	**0.3 nm**	**S1** C-1	**S2** S(C-1)	**S3** C-2	**S4** S(C-2)
peak position [BE, eV]	284.4	286.2	285.6	287.7		284.6	286.4	285.8	287.9
LW [eV]	0.26	0.26	0.26	0.26		0.24	0.24	0.24	0.24
GW [eV]	1.00	1.00	1.00	1.00		1.26	1.26	1.26	1.26
rel. area [%]	65.0	10.1	20.9	4.0		65.0	10.1	20.9	4.0

6. N-K edge XAS spectrum of a 5.5 nm thick FePc film on rutile TiO_2(110)

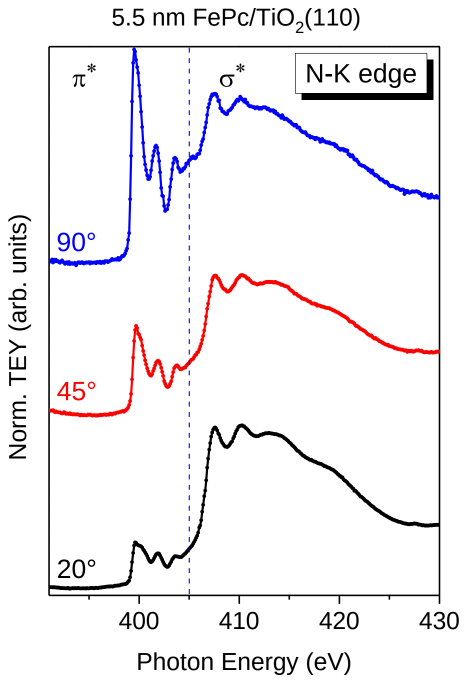

Figure S4: N-K edge X-ray absorption spectra for a thick FePc film on rutile TiO_2(110) (prepared in presence of oxygen during the last annealing step). The maximum of π* resonances is observed at normal incidence (90°), indicating a preferred standing orientation of FePc molecules.

7. XPS spectra of FePc on rutile TiO_2(110) prepared in presence of oxygen

Table S6: Peak fit parameters for C 1s and N 1s spectra of a 7.3 nm and a 0.2 nm thick FePc film on rutile TiO_2(110) (manuscript, Figure 10). Top: N 1s, bottom: C 1s

**7.3 nm**	**S1** N-1	**S2** S(N-1)	**0.2 nm**	**S1** N-1	**S2** NE-1	**S3** S(N-1)
peak position [BE, eV]	398.8	400.4		399.0	400.9	400.6
LW [eV]	0.46	0.46		0.46	0.46	0.46
GW [eV]	1.11	1.11		1.24	1.24	1.24
rel. area [%]	94	6		90	5	5

**7.3 nm**	**S1** C-1	**S2** S(C-1)	**S3** C-2	**S4** S(C-2)	**0.2 nm**	**S1** C-1	**S2** S(C-1)	**S3** C-2	**S4** S(C-2)
peak position [BE, eV]	284.4	286.2	285.6	287.7		284.7	286.5	285.8	288.0
LW [eV]	0.26	0.26	0.26	0.26		0.26	0.26	0.26	0.26
GW [eV]	0.98	0.98	0.98	0.98		1.26	1.26	1.26	1.26
rel. area [%]	65.0	10.1	20.9	4.0		65.0	10.1	20.9	4.0

8. XPS spectra of FePc on rutile TiO2(110) prepared in absence of oxygen

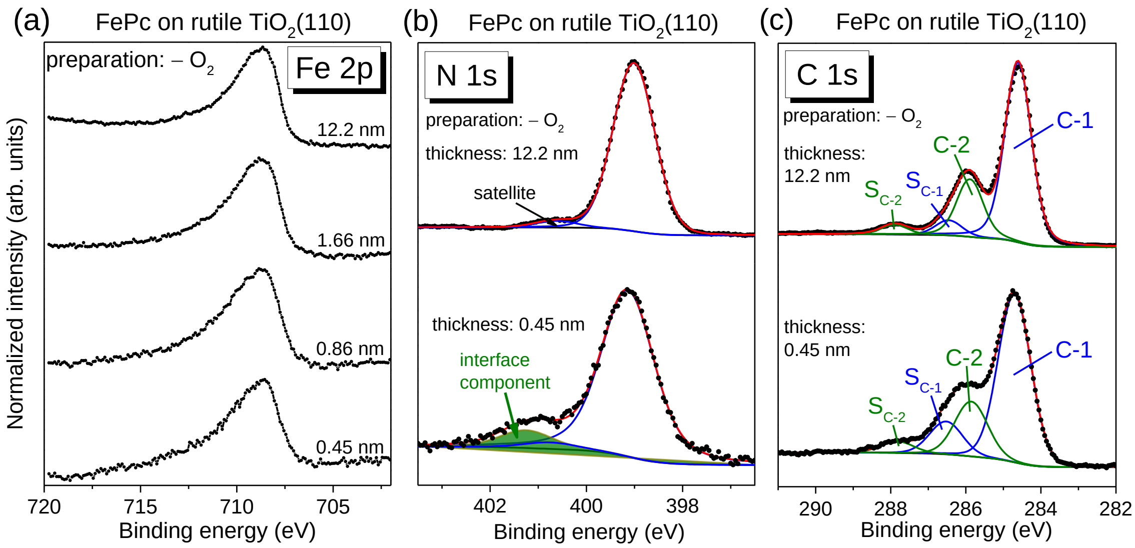

Figure S5: Thickness dependent core level spectra of FePc on rutile $TiO_2(110)$ (excited with monochromatized Al Kα radiation, hν = 1486.6 eV): (a) Fe 2p, (b) N 1s, (c) C 1s. Similar to FePc on rutile $TiO_2(100)$, the spectra are essentially broadened for low coverages and a weak interface component is found in N 1s spectra. Note that the fitting parameters differ from measurements with conventional Al Kα excitation, in particular with respect to the Gaussian width.

Table S7: Peak fit parameters of spectra shown in Figure S5.

**12.2 nm**	**S1** N-1	**S2** S(N-1)	**0.45 nm**	**S1** N-1	**S2** NE-1	**S3** S(N-1)
peak position [BE, eV]	398.8	400.4		399.2	401.2	400.8
LW [eV]	0.10	0.10		0.10	0.10	0.10
GW [eV]	0.96	0.96		1.26	1.26	1.26
rel. area [%]	96	4		86	11	3

**12.2 nm**	**S1** C-1	**S2** S(C-1)	**S3** C-2	**S4** S(C-2)	**0.45 nm**	**S1** C-1	**S2** S(C-1)	**S3** C-2	**S4** S(C-2)
peak position [BE, eV]	284.6	286.5	285.9	287.9		284.7	286.5	285.9	287.8
LW [eV]	0.10	0.10	0.10	0.10		0.10	0.10	0.10	0.10
GW [eV]	0.81	0.81	0.81	0.81		0.97	0.97	0.97	0.97
rel. area [%]	68.2	6.0	22.3	3.5		62.8	12.3	20.6	4.3

9. Ultraviolet–visible (UV-vis) absorption spectra of FePc and $FePcF_{16}$

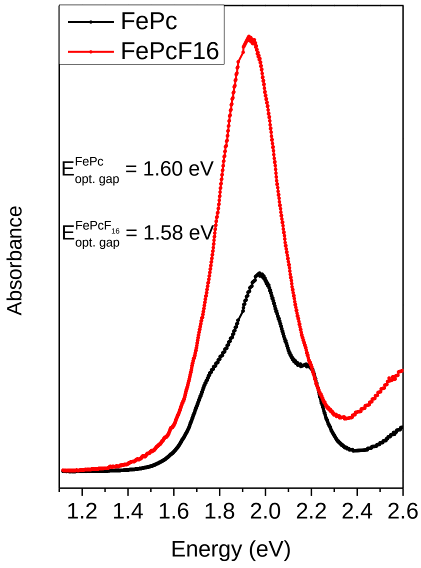

Figure S6: UV-vis spectra of FePc (black) and $FePcF_{16}$ (red) evaporated on glass. The optical band gap was determined by extrapolation of the onset of the spectra. We obtain 1.60 eV and 1.58 eV for FePc and $FePcF_{16}$, respectively.

10. Thickness dependent Fe 2p XPS spectra of $FePcF_{16}$ on rutile $TiO_2(100)$

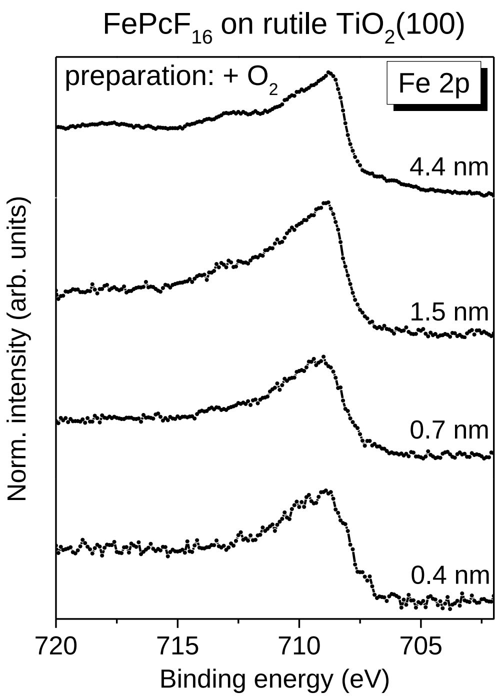

Figure S7: Thickness dependent Fe 2p core level spectra of $FePcF_{16}$ on rutile $TiO_2(100)$ (excited with monochromatized Al Kα radiation, hν = 1486.6 eV), prepared in presence of oxygen during the last annealing step. No interface component could be detected. The different peak shape, compared to FePc, might be due to a different spin configuration of Fe in thin films.

11. N 1s and C 1s peak fit parameters of $FePcF_{16}$ on rutile $TiO_2(100)$

Table S8: Peak fit parameters of a 4.4 nm and a 0.4 nm thick $FePcF_{16}$ film on rutile $TiO_2(100)$ (manuscript, Figure 11), which is prepared in an oxygen partial pressure and measured with a monochromatized X-ray source (1486.6 eV). (a) N 1s, (b) C 1s 4.4 nm thick film, (c) C 1s 0.4 nm thick film.

**(a) 4.4 nm**	**S1** N-1	**S2** S(N-1)	**0.4 nm**	**S1** N-1	**S2** NE-1	**S3** S(N-1)
peak position [BE, eV]	399.2	400.8		399.2	401.5	400.8
LW [eV]	0.10	0.10		0.10	0.10	0.10
GW [eV]	0.98	0.98		1.25	1.25	1.25
rel. area [%]	95	5		86	10	4

**(b) 4.4 nm**	**S1** C-1	**S2** S(C-1)	**S3** C-2	**S4** S(C-2)	**S5** C-3	**S6** S(C-3)
peak position [BE, eV]	284.9	286.7	286.0	287.9	287.0	288.7
LW [eV]	0.17	0.17	0.17	0.17	0.17	0.17
GW [eV]	0.81	0.81	0.81	0.81	0.81	0.81
rel. area [%]	23.4	1.3	17.0	6.8	45.8	5.7

**(c) 0.4 nm**	**S1** C-1	**S2** S(C-1)	**S3** C-2	**S4** S(C-2)	**S5** C-3	**S6** S(C-3)
peak position [BE, eV]	285.3	287.1	286.3	288.2	287.4	289.1
LW [eV]	0.20	0.20	0.20	0.20	0.20	0.20
GW [eV]	0.91	0.91	0.91	0.91	0.91	0.91
rel. area [%]	23.5	1.1	17.0	6.7	46.0	5.7

12. Peak fit parameters of the Ti 2p spectrum shown in Figure 12(a)

Table S9: Ti 2p peak fit parameters of rutile $TiO_2(100)$, prepared in UHV and measured with a monochromatized X-ray source (1486.6 eV). The defect-rich preparation makes it necessary to consider an extra component for Ti^{2+}.

FePcF16/TiO_2(100) without O_2, hexagonal	**D1 (Ti^{4+})** Component1 Component2	**D2 (Ti^{3+})** Component1 Component2	**D4 (Ti2+)** Component1 Component2
peak position [BE, eV]	459.34<br>464.93	457.54<br>463.13	456.30<br>461.89
LW [eV]	1.00<br>1.80	1.00<br>1.80	1.00<br>1.80
GW [eV]	1.03<br>1.48	1.03<br>1.48	1.03<br>1.48
rel. area [%]	63	30	7

13. Peak fit parameters of $FePcF_{16}$ on defect-rich rutile $TiO_2(100)$ prepared without O_2

The parameters are related to Figure 13 of the manuscript. The excitation source was a monochromatized X-ray source (1486.6 eV).

13.1. N 1s

Table S10: N 1s peak fit parameters for a 5.6 nm, a 0.6 nm and a 0.3 nm thick $FePcF_{16}$ film.

**5.6 nm**	**S1** N-1	**S2** S(N-1)	**0.6 nm**	**S1** N-1	**S2** NE-1	**S3** S(N-1)	**0.3 nm**	**S1** N-1	**S2** NE-1	**S3** S(N-1)
peak position [BE, eV]	398.8	400.4		399.2	400.8	400.8		399.3	400.9	400.9
LW [eV]	0.10	0.10		0.10	0.10	0.10		0.10	0.10	0.10
GW [eV]	1.19	1.19		1.47	1.47	1.47		1.47	1.47	1.47
rel. area [%]	95	5		80	16	4		65	31	4

13.2. C 1s (film thickness 5.6 nm)

Table S11: C 1s peak fit parameter of the 5.6 nm thick $FePcF_{16}$ film.

**5.6 nm**	**S1** C-1	**S2** S(C-1)	**S3** C-2	**S4** S(C-2)	**S5** C-3	**S6** S(C-3)
peak position [BE, eV]	284.8	286.6	285.9	287.8	286.9	288.6
LW [eV]	0.17	0.17	0.17	0.17	0.17	0.17
GW [eV]	0.78	0.78	0.78	0.78	0.78	0.78
rel. area [%]	23.1	1.5	17.5	6.2	46.1	5.6

13.3. C 1s (film thickness 0.6 nm)

Table S12: C 1s peak fit parameter for the 0.6 nm thick $FePcF_{16}$ film. (a) First set of carbon components (C-1, C-2, C-3 and their satellites). (b) Set of additional components (CE-1, CE-2, CE-3 and their satellites). The Gauss and Lorentz peak widths and the peak distances are kept constant for both set of components.

**(a) 0.6 nm**	**S1**<br>C-1	**S2**<br>S(C-1)	**S3**<br>C-2	**S4**<br>S(C-2)	**S5**<br>C-3	**S6**<br>S(C-3)
peak position [BE, eV]	285.0	286.8	285.9	288.0	287.4	288.8
LW [eV]	0.17	0.17	0.17	0.17	0.17	0.17
GW [eV]	1.1	1.1	1.1	1.1	1.1	1.1
rel. area [%]	19.1	1.2	14.5	5.1	38.2	4.6

**(b) 0.6 nm**	**S7**<br>CE-1	**S8**<br>S(CE-1)	**S9**<br>CE-2	**S10**<br>S(CE-2)	**S11**<br>CE-3	**S12**<br>S(CE-3)
peak position [BE, eV]	286.2	288.0	287.1	289.2	288.6	290
LW [eV]	0.17	0.17	0.17	0.17	0.17	0.17
GW [eV]	1.1	1.1	1.1	1.1	1.1	1.1
rel. area [%]	4.0	0.3	3.1	1.1	8.1	0.7

13.4. C 1s (film thickness 0.3 nm)

Table S13: C 1s peak fit parameters for the 0.3 nm $FePcF_{16}$ on defect-rich TiO2(100). (a) First set of carbon components (C-1, C-2, C-3 and their satellites). (b) Set of extra components (CE-1, CE-2, CE-3 and their satellites). The Gauss and Lorentz peak widths and the peak distances are kept constant for both set of components.

**(a) 0.3 nm**	**S1**<br>C-1	**S2**<br>S(C-1)	**S3**<br>C-2	**S4**<br>S(C-2)	**S5**<br>C-3	**S6**<br>S(C-3)
peak position [BE, eV]	285.2	287.0	286.1	288.2	287.6	289
LW [eV]	0.17	0.17	0.17	0.17	0.17	0.17
GW [eV]	1.1	1.1	1.1	1.1	1.1	1.1
rel. area [%]	17.1	1.1	13.0	4.6	34.2	4.1

**(b) 0.3 nm**	**S7**<br>CE-1	**S8**<br>S(CE-1)	**S9**<br>CE-2	**S10**<br>S(CE-2)	**S11**<br>CE-3	**S12**<br>S(CE-3)
peak position [BE, eV]	286.5	288.3	287.4	289.5	288.9	290.3
LW [eV]	0.17	0.17	0.17	0.17	0.17	0.17
GW [eV]	1.1	1.1	1.1	1.1	1.1	1.1
rel. area [%]	6.0	0.4	4.5	1.6	11.9	1.5

13.5. F 1s

Table S14: F 1s peak fit parameters of spectra shown in Figure 13.

(a) 5.6 nm	S1<br>F-1	S2<br>S(F-1)	S3<br>F-2
peak position [BE, eV]	687.2	688.7	684.0
LW [eV]	0.23	0.23	0.23
GW [eV]	1.28	1.28	1.28
rel. area [%]	88.1	7.2	4.7

(b) 0.6 nm	S1<br>F-1	S2<br>S(F-1)	S3<br>F-2	S4<br>FE-1
peak position [BE, eV]	687.7	689.2	684.8	686.6
LW [eV]	0.23	0.23	0.23	0.23
GW [eV]	1.47	1.47	1.47	1.47
rel. area [%]	73.6	6.0	13.2	7.2

(c) 0.3 nm	S1<br>F-1	S2<br>S(F-1)	S3<br>F-2	S4<br>FE-1
peak position [BE, eV]	687.9	689.4	684.9	686.8
LW [eV]	0.23	0.23	0.23	0.23
GW [eV]	1.47	1.47	1.47	1.47
rel. area [%]	63.6	5.2	16.2	15.0

14. Fe 2p XPS spectra of $FePcF_{16}$ on defect-rich rutile $TiO_2(100)$ prepared without O_2

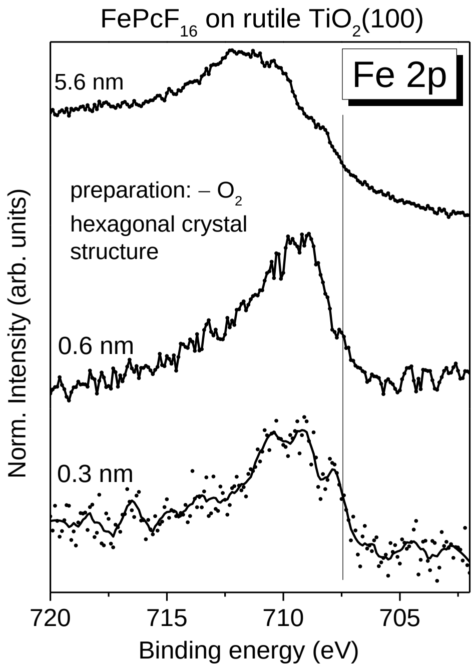

Figure S8: Thickness dependent Fe 2p core level spectra of $FePcF_{16}$ on rutile $TiO_2(100)$ (excited with monochromatized Al Kα radiation, hν = 1486.6 eV), prepared in absence of oxygen. Extra intensity in monolayer spectrum at 707.5 eV (black line in S8) may point to an interface component. The broadening for high film thickness might be caused by a change of the Fe spin state in $FePcF_{16}$.
